# Public preferences for the value and implementation of genomic newborn screening: Insights from two discrete choice experiments in Australia

**DOI:** 10.1016/j.ajhg.2025.05.001

**Published:** 2025-05-28

**Authors:** Riccarda Peters, Stephanie Best, Fiona Lynch, Danya F. Vears, Lilian Downie, Alison D. Archibald, Sebastian Lunke, Zornitza Stark, Ilias Goranitis

**Affiliations:** 1 Economics of Genomics and Precision Medicine Unit, Centre for Health Policy, Melbourne School of Population and Global Health, The University of Melbourne, Melbourne, VIC 3052, Australia; 2 Australian Genomics, Melbourne, VIC 3052, Australia; 3 Melbourne School of Health Sciences, Faculty of Medicine, Dentistry and Health Sciences, The University of Melbourne, Melbourne, VIC 3052, Australia; 4 Biomedical Ethics Research Group, Murdoch Children’s Research Institute, Parkville, VIC, Australia; 5 Melbourne Law School, The University of Melbourne, Melbourne, VIC 3052, Australia; 6 Department of Paediatrics, Faculty of Medicine, Dentistry and Health Sciences, The University of Melbourne, Melbourne, VIC 3052, Australia; 7 Victorian Clinical Genetics Services, Murdoch Children’s Research Institute, Parkville, VIC 3052, Australia; 8 Genomics in Society, Murdoch Children’s Research Institute, Parkville, VIC, Australia; 9 Department of Clinical Pathology, Faculty of Medicine, Dentistry and Health Sciences, The University of Melbourne, Melbourne, VIC 3052, Australia

**Keywords:** newborn screening, health economics, genomic sequencing, genomic newborn screening, public views, discrete choice experiment, DCE, societal preferences

## Abstract

Integrating genomic sequencing into newborn screening (NBS) has transformative potential for the identification and management of genetic conditions. Using discrete choice experiment surveys, we elicited the preferences, values, and priorities of 2,509 members of the Australian public about the value (*n* = 1,504) and implementation (*n* = 1,005) of genomic NBS (gNBS). The Australian public demonstrated positive preference for gNBS, with 90% of respondents indicating an interest in gNBS results. Cost of screening was the most important attribute in people’s decision about uptake of gNBS. Enabling diagnosis in more newborns increases the utility of gNBS. To enable these diagnoses, the public is willing to accept less restrictive models of gNBS in terms of the types of conditions included. However, there is disutility associated with including conditions that have less effective (or no) treatments available and including conditions with reduced penetrance. A gNBS program yielding 10–50 additional diagnoses per 1,000 newborns screened relative to standard NBS was valued by the Australian public at AU$4,600–$5,700 (US$2,990–$3,700) per newborn screened. Most participants (65%) preferred an opt-in type of consent and expressed a preference to receive high-chance results in person from a genetics professional, although telehealth and phone options were acceptable. Our findings should inform economic evaluation and future implementation for gNBS in the Australian and other healthcare systems.

## Teaser

Genomics will potentially transform newborn screening programs globally. To inform implementation, we surveyed 2,509 members of the Australian public to understand the importance they place on key aspects of genomic newborn screening and their preferences about how programs should be delivered in practice.

## Introduction

Newborn screening (NBS) is a public health success story.^1^ NBS programs reduce morbidity and mortality through the early identification and management of serious but treatable conditions that benefit from early intervention.^2^ NBS programs are implemented in many countries and achieve near-universal uptake.^2^ Traditional NBS programs primarily measure biochemical markers in blood collected on blood spot cards within the first 48 h of life using mass spectrometry. While this method is highly accurate, it limits detection to conditions where a biochemical marker is available. New technologies can be used in NBS to look for many more health conditions. One of these technologies is genomic sequencing, which has the potential to revolutionize NBS programs by analyzing hundreds of genes associated with rare diseases simultaneously.^3^ Though individually rare, these conditions collectively represent a substantial health and economic burden on the population and health systems.^4^ Currently there are many large-scale cohort studies under way internationally generating evidence to guide the implementation of genomic sequencing into NBS programs, commonly referred to as genomic newborn screening (gNBS).^3^

While gNBS offers many opportunities, such as earlier and faster identification of a much wider range of conditions, there are also considerable challenges that need to be addressed if it were implemented as a population-level screening program.^5^^,^^6^ Importantly, identifying pathogenic variants through genomic screening does not equate to a clinical diagnosis,^3^ and robust evidence is needed regarding its clinical utility, accuracy, and impact on health outcomes.^5^^,^^6^ There are also ethical, legal, and social considerations of gNBS including balancing the best interests of the child and family, selecting appropriate genes, the possibility of secondary or incidental findings, and ensuring equitable access and outcomes.^7^ The possible implementation of gNBS at scale requires careful consideration to address both the ethical and practical aspects,^5^^,^^6^^,^^8^ and care must also be taken to maintain high uptake.^9^

Existing NBS programs already take variable approaches to the number of conditions screened and how the service is delivered, such as the amount of information provided to parents or the need for explicit consent.^2^ The generation of genomic data will likely amplify these issues.^3^^,^^5^ Notable variations can be observed within the gNBS pilot studies currently in progress, such as decisions on which conditions to include,^10^^,^^11^^,^^12^^,^^13^ placing variable emphasis on analytical and clinical validity, penetrance, age at condition onset, and actionability.^3^ However, harmonization efforts are emerging.^13^ From an implementation perspective, to incorporate genomic technologies in NBS, the whole screening pathway will need to be modified, including changes from how information is delivered to parents to support informed decision-making to how data are stored.^3^ An important component of ensuring successful and value-based implementation of gNBS is understanding public preferences, values, and priorities.

Discrete choice experiments (DCEs) represent a survey-based method widely used to determine individual preferences for health, healthcare services, technologies, and other goods and services influencing health.^14^ DCEs are a valuable tool for eliciting preferences and quantifying how individuals and the public trade off various attributes of health interventions.^15^ There is an increasing application of DCEs in the context of genomics,^16^^,^^17^ and preferences have been used to inform economic evaluations in this context.^17^^,^^18^^,^^19^^,^^20^^,^^21^^,^^22^^,^^23^^,^^24^

This study reports on two DCEs conducted as part of the BabyScreen+ program to elicit the Australian public’s preferences, values, and priorities for gNBS and its implementation. The first DCE aimed to assess the value of gNBS (value DCE), while the second examined preferences regarding service delivery (implementation DCE), including when and how information about gNBS should be delivered and how results should be returned. The findings provide critical insights into the Australian public preferences and values for gNBS, and its translation and will enable cost-benefit evaluations to support the sustainable implementation of genomics into health systems.

## Material and methods

### Study design and participants

A DCE survey is a research method used to understand patient or healthcare provider preferences by presenting them with a series of hypothetical scenarios. In each scenario, respondents are asked to choose between different options, each characterized by a set of features or “attributes” (e.g., treatment effectiveness and cost). These attributes have different “levels” (e.g., high vs. low effectiveness). By analyzing the choices people make, DCE surveys help to identify the relative importance of each attribute and the trade-offs respondents are willing to make between them.^18^

The DCE surveys were designed following best practice recommendations for development,^19^ analysis,^18^ and reporting.^14^ As recommended,^20^ focus groups were conducted to identify and develop the DCE attributes, and detailed information about the focus groups’ methods and findings is available elsewhere.^21^^,^^22^ Attribute levels were identified in consultation with the genetic experts of the research team to ensure clinical face validity. The final attributes and corresponding levels and descriptions of both DCEs are shown in Tables 1 and 2.

**Table 1:** Attributes and attribute levels included in the value discrete choice experiment (DCE)

**Attributes**	**Definition**	**Levels**
Severity of conditions (without treatment)	how severe the conditions included in the genomic newborn screening program are in the absence of treatment. Severity was defined based on impact on quality of life and life expectancy, ranging from minimal to significant effects on both dimensions	1. Profound only
		2. Profound and moderate
		3. Profound, moderate, and mild
Certainty that the condition develops (without treatment)	information about how likely it is that the condition develops. If genomic newborn screening shows a high chance that a child might develop a condition, it is not certain the child will end up developing the condition. For example: high certainty means that medical experts are very confident (90%) that the condition will develop	1. High, moderate, and average certainty (greater than 50%)
		2. High and moderate certainty (greater than 75%)
		3. High certainty only (greater than 90%)
Treatment availability for conditions screened	indicates whether there are ways to treat the conditions diagnosed from genetic newborn screening. Some conditions can be treated to either cure them or manage their symptoms. For others, there are no treatments available at the moment	1. Treatments that cure conditions
		2. Treatments that cure conditions or manage their symptoms
		3. Treatments that cure conditions or manage their symptoms and conditions without current treatment
Additional number of newborns diagnosed compared to standard newborn screening (in every 1,000 newborns screened)	additional number of newborns that will be identified with a high chance of developing a condition through genomic newborn screening compared to standard newborn screening	1. 3 in 1,000
		2. 10 in 1,000
		3. 20 in 1,000
		4. 50 in 1,000
Accuracy of screening results	how likely it is that the screening results are correct. Capturing the overall reliability of the screening results	1. 95% (5 out of 100 screened newborns will receive a wrong initial diagnosis)
		2. 98% (2 out of 100 screened newborns will receive a wrong initial diagnosis)
		3. 100% (none of the newborns will receive a wrong initial diagnosis)
Cost of genomic newborn screening to you	because genomic newborn screening is a new program, the Federal Government may not pay for it. This characteristic tells you how much you would need to pay out-of-pocket for genomic newborn screening	1. $500
		2. $1,000
		3. $2,500

**Table 2:** Attributes and attribute levels included in the implementation DCE

**Attributes**	**Levels**
When is genomic newborn screening first discussed?	early during pregnancy at first doctor appointment
	second trimester
	third trimester
	shortly after birth
Who provides initial information?	a midwife or nurse
	your GP
	an obstetrician
	a genetic health professional
What support material is available?	no support material
	leaflet
	interactive online portal
	appointment with health professional
What conditions are included?	conditions selected by parents
	conditions selected by health professionals
Who returns “high-chance” results?	your GP
	a relevant medical specialist
	a genetic health professional
How are “high-chance” results returned?	electronically through a secure online portal
	directly, telehealth, or phone
	directly, in person
How are “low-chance” results returned?	no return of “low-chance” results
	electronically through a secure online portal
	directly, telehealth, or phone
	directly, in person
What happens if new relevant information becomes available?	no updates will be provided
	updates will be provided upon request
	updates will be provided automatically in a secure online portal

**Figure 1:**
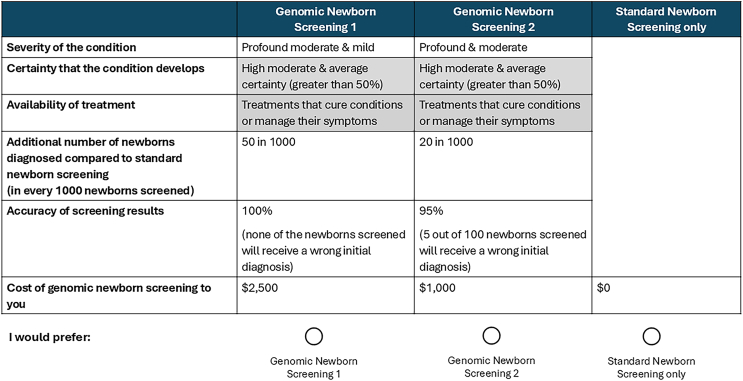
Choice task example for the value DCE

**Figure 2:**
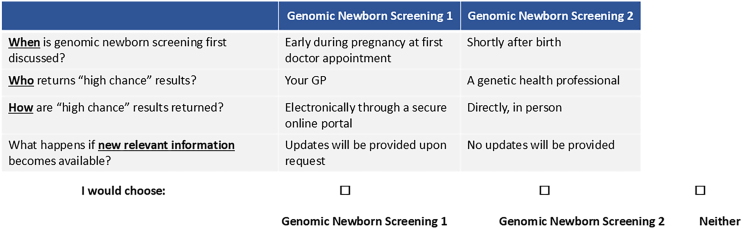
Choice task example for the implementation DCE

To ensure participants were adequately prepared with information to complete the surveys, the surveys included an educational component, which comprised a video developed by the BabyScreen+ study team to explain gNBS. Participants were further presented with an explanation of gNBS characteristics and were guided through an explanation of the DCE with an example. Participants were then presented with a choice scenario involving two gNBS alternatives and an opt-out option representing the standard NBS, as shown in Figures 1 and 2, respectively. Choice scenarios were designed using a labeled D-efficient partial profile design in Ngene (ChoiceMetrics [2024] Ngene 1.4 User Manual and Reference Guide, Australia, https://www.choice-metrics.com/NgeneManual140.pdf), which helps reduce task complexity. The design for the value DCE included 32 choice tasks split across four blocks, while 64 choice tasks were split across six blocks for the implementation DCE. Thus, for both surveys each participant was required to complete eight choice tasks. Full details about the experimental design used in the surveys are provided in the supplemental methods.

The surveys were developed with support from a plain-language advisor and piloted using think-aloud interviews among a convenience sample of university staff to assess completion time, language, and understanding (*n* = 5). The surveys underwent several rounds of piloting (*n* = 873) to evaluate whether the coefficients were logically ordered, to assess completion time, and to gather feedback on the difficulty of the task. The pilot results were used to refine the DCE experimental design and final survey wording. The final surveys are available in the supplemental information.

In accordance with recommended practices on the measurement and valuation of health benefits for economic evaluation,^23^ we sought the values of the general public, who are both taxpayers and potential users of healthcare. Two independent Australia-wide samples of participants over the age of 18 years were recruited from nationwide panels through the research market company Pureprofile. Age, gender, income, and geographical location (states) quotas were used to ensure that the sample was representative of the Australian public. This was further validated against other national sources.^24^

### Ethics statement

Informed consent was obtained from all participants before entering the survey. Ethics approval was granted from the Royal Children’s Hospital Melbourne Human Research Ethics Committee (Ethics ID: HREC/91392/RCHM-2022).

### Choice analysis

Choice data were analyzed using a panel error component mixed logit model, which uses random parameters to account for unobserved heterogeneity of preferences among participants.^18^ Full information about the coding of the attributes and analytical methods used in choice analysis is provided in the supplemental methods. We estimate the relative importance of each attribute based on the proportional change in overall utility associated with transitioning from the lowest to the highest level of each attribute.^25^ We further explore preference heterogeneity across both DCEs using a variety of methods including a latent class choice model, which divides the sample into a finite number of groups (classes) with homogeneous preferences. Full information on the econometric analysis of our choice data is available in the supplemental methods. The results of the latent class model were used to provide an estimate of the uptake of gNBS based on the proportion of participants demonstrating no preference for gNBS and its attributes.

Finally, we estimate the overall value that the public attaches to a publicly funded gNBS with a restrictive and non-restrictive gNBS implementation relative to standard NBS using the compensating variation method.^26^ The restrictive gNBS model includes conditions of profound severity, high chance of developing (>90%), and curative treatments available. The non-restrictive gNBS model includes conditions of profound, moderate, and mild severity, conditions with >50% chance of developing, conditions with curative treatments available, and treatments to manage symptoms and conditions without current treatment. The values are reported in both Australian and US dollars (using November 21, 2024 Reserve Bank of Australia exchange rate of 0.65). Analyses were performed in Nlogit 6 (Econometric Software, Waverton, NSW, Australia) and Stata (StataCorp, College Station, TX, USA).

## Results

### Demographics

Overall, 2,509 members of the Australian public participated in the value (*n* = 1,504) and implementation (*n* = 1,005) DCEs. The samples were similar in terms of age, gender, household income, and geographical location compared to the national census summary.^24^ Detailed information about the socioeconomic status, demographics, and responses regarding experience with genomics and knowledge about genetic conditions and newborn screening is provided in Table S1.

Across the two surveys, 16% of respondents had experience with a genetic condition, 20% had experience with genetic or genomic testing, 42% had heard about genomic testing before the survey, and about half of respondents knew a little (43%) to a lot (6%) about newborn screening before participating.

A total of 1,504 participants were asked to indicate their preference for which conditions should be included in gNBS based on age of onset of the condition. Multiple selections were allowed. The majority indicated a preference for conditions with an onset in infancy (72%) and early childhood (49%) (Table S2). About 30% of respondents indicated preference for conditions with onset in childhood and adolescence and 23% for adult-onset conditions, with 10% of respondents indicating no preference for gNBS. Another group of 1,005 participants were asked to indicate their preferred method of consent for gNBS. The majority of participants (65%) preferred an opt-in consent process (Table S2).

### Value DCE results and interpretation

**Figure 3:**
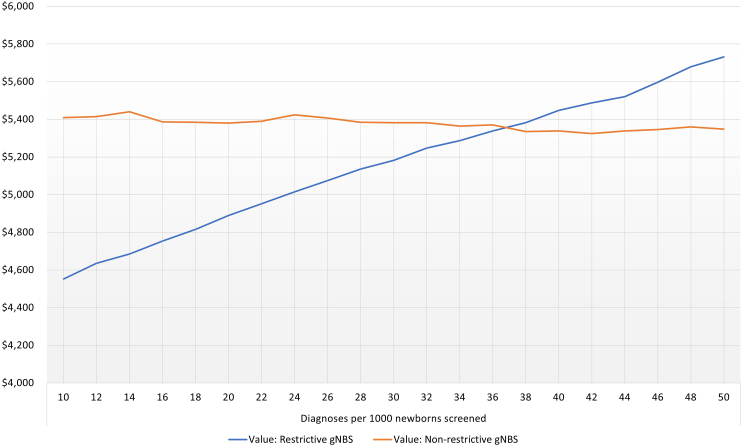
The incremental value in Australian dollars of publicly funded genomic newborn screening (gNBS) applications relative to standard NBS as a function of the additional diagnoses enabled per 1,000 newborns screened Restrictive gNBS includes conditions of profound severity, high chance of developing (>90%), and curative treatments available; non-restrictive gNBS includes conditions of profound, moderate, and mild severity, conditions with >50% chance of developing, conditions with curative treatments available and treatments to manage symptoms, and conditions without current treatment.

The results of the Value DCE are presented in Table 3. The members of the public demonstrated statistically significant preferences across all gNBS attributes. Cost of testing was the most significant driver in people’s choice to have gNBS or not. Accuracy in screening results also increases the utility for gNBS. Enabling diagnosis in more newborns was an important driver of respondents’ utility for gNBS, and, as such, respondents on average were willing to accept gNBS models that included conditions of moderate and mild severity, conditions with moderate and average certainty of developing, and conditions with treatments to manage symptoms as well as conditions without current treatment available. However, when controlling for the number of diagnoses achieved through restrictive and less restrictive gNBS models, it is evident that respondents preferred more restrictive gNBS models (i.e., inclusion of conditions with profound severity only, high certainty of developing, and curative treatments available). This means that as more diagnoses are enabled through gNBS, the value of more conservative gNBS models is increasing, while the value of less restrictive models remains relatively stable given that the added utility from new diagnoses is mitigated by the disutility of having less effective (or no) treatments available and greater uncertainty about whether a condition will develop. As shown in Figure 3, the value of a restrictive gNBS model increases from AU$4,600 (US$2,990) to $5,700 (US$3,700) per newborn screened as the incremental screening yield of gNBS relative to standard NBS increases from 10 to 50 diagnoses per 1,000 newborns screened, whereas the value of a non-restrictive gNBS model remains relatively stable at AU$5,400 (US$3,510) per newborn screened.

**Table 3:** Attribute marginal utilities and importance scores for the value DCE

	**Attributes**	**Mean**	**SE**	**SD**	**SE**	**Importance score (%)**	**Ranking**
Severity	profound conditions only	base	–	–	–	16.4%	2
	profound and moderate conditions	0.85198^a^	0.1537	0.57285	0.3419	–	–
	profound, moderate, and mild conditions	1.35147^a^	0.1372	1.44105^a^	0.13944	–	–
Certainty	high, moderate, and average certainty (greater than 50%)	base	–	–	–	8.9%	6
	high and moderate certainty (greater than 75%)	−0.36018^a^	0.1671	0.94002^a^	0.3009	–	–
	high certainty only (greater than 90%)	−0.73117^a^	0.1103	0.62383^a^	0.1597	–	–
Treatment availability	treatments that cure conditions	base	–	–	–	11.2%	5
	treatments that cure conditions or manage their symptoms	0.51008^a^	0.1221	0.00518	0.4171	–	–
	treatments that cure conditions or manage their symptoms and conditions without current treatment	0.92122^a^	0.1216	1.20558^a^	0.12109	–	–
Additional number of newborns diagnosed compared to standard newborn screening (in every 1,000 newborns screened)	–	0.0266^a^	0.0060	0.0635^a^	0.0031	15.2%	4
Accuracy of screening results	95%	base	–	–	–	16.2%	3
	98%	0.83427^a^	0.0644	0.31191	0.17916	–	–
	100%	1.331^a^	0.0668	1.25738^a^	0.0765	–	–
Cost of testing to you (AU$)	–	−0.00132^a^	0.5301D-04	0.00132^a^	0.5301D-04	32.1%	1
Genomic newborn screening constant	–	4.97359^a^	0.3318	6.36818^a^	0.3192	–	–

**Interactions**							

Additional diagnoses × profound and moderate conditions	–	−0.01599^a^	0.0047	0.00319	0.123	–	–
Additional diagnoses × profound, moderate, and mild conditions	–	−0.01403^a^	0.0054	0.3187^a^	0.0054	–	–
Additional diagnoses × high and moderate certainty (greater than 75%)	–	−0.01029^a^	0.0046	0.00669	0.01028	–	–
Additional diagnoses × high certainty only (greater than 90%)	–	0.01744^a^	0.0051	0.1209	0.0090	–	–
Additional diagnoses × treatments that cure conditions or manage their symptoms	–	−0.00673	0.0050	0.01661^a^	0.00683	–	–
Additional diagnoses × treatments that cure conditions or manage their symptoms and conditions without current treatment	–	−0.01735^a^	0.0047	0.00713	0.0078	–	–
Log likelihood function	−8183.22114	–	–	–	–	–	–
McFadden pseudo *R*-squared	0.3809268	–	–	–	–	–	–
Akaike information criterion	16434.4	–	–	–	–	–	–

SE, standard error; SD, standard deviation.

Regression coefficients show the marginal impact of each attribute (or attribute level) on the utility of genomic newborn screening. Positive (or negative) mean estimates suggest an average positive (or negative) effect on utility. SD estimates illustrate the variability in preferences among the study participants.

a Statistically significant at 1% level.

The standard deviations for most parameters were statistically significant, indicating heterogeneity of preferences among respondents (Table 3). The latent class analysis revealed four groups (classes) of participants with more homogeneous preferences (Table S3). Class 1 corresponds to people who had a negative preference for gNBS, evidenced by the high negative constant, and made up 13% of the overall sample. However, the remaining 87% had a positive preference for gNBS. Class 2 comprises 28% of the sample and includes people with positive preference for testing who demonstrated disutility for higher costs and utility for screening accuracy. Classes 3 and 4, comprising 42% and 17% of the sample, were distinguished based on how they valued different attributes: cost and number of diagnoses were not significant drivers of choice-making for class 3 but were major choice determinants in class 4. Class 1 participants tended to be of higher age, lower education, and lower income. They also tended to have had children, had less experience with genetic conditions, and had less knowledge about genetic conditions (Table S4). Class 3 participants tended not to have children, had experience with and had knowledge about genetic conditions, and had higher health literacy.

### Results of implementation DCE

The results of the implementation DCE are shown in Table 4. Participants exhibited greater utility for receiving initial information about the screening program from their general practitioner (GP) or obstetrician. Participants demonstrated preference for receiving results that indicate a high chance for their child having a genetic condition (high-chance result) from a genetics health professional either in person or through telehealth or phone. Even low-chance results, when screening does not detect any increased chance of developing the medical problems screened for, were preferred to be communicated in person rather than electronically.

**Table 4:** Attribute marginal utilities, importance scores, and ranking for the implementation DCE

**Attribute**	**Level**	**Mean**	**SE**	**SD**	**SE**	**Importance score (%)**	**Ranking**
When is gNBS first discussed?	first doctor appointment	base	–	–	–	9.5%	6
	second trimester	0.07769	0.0802	0.34563^b^	0.135	–	–
	third trimester	0.11337	0.07463	0.13863	0.334	–	–
	shortly after birth	0.12099	0.07505	0.0129	1.497	–	–
Who provides initial information?	a midwife or nurse	base	–	–	–	13.2%	2
	your GP	0.16861^b^	0.07363	0.06516	0.624	–	–
	an obstetrician	0.14897^b^	0.07194	0.03184	1.071	–	–
	a genetic health professional	0.08439	0.06126	0.19297	0.187	–	–
What support material is available?	no support material	base	–	–	–	9.9%	5
	leaflet	0.12639	0.07	0.06516	1.100	–	–
	interactive online portal	0.09817	0.07619	0.03184	0.212	–	–
	appointment with health professional	0.11875	0.07538	0.19297	0.176	–	–
What conditions are included?	selected by parents	base	–	–	–	9.1%	7
	selected by health professionals	0.11541	0.08652	0.56115^b^	0.237	–	–
Who returns “high-chance” results?	your GP	base	–	–	–	12.5%	4
	a relevant medical specialist	0.12933	0.0799	0.28424	0.241	–	–
	a genetic health professional	0.15933^b^	0.07224	0.42372^a^	0.154	–	–
How are “high-chance” results returned?	online portal	base	–	–	–	24.0%	1
	telehealth or phone	0.26060^a^	0.08248	0.30187	0.225	–	–
	in person	0.30525^a^	0.073	0.32142	0.200	–	–
How are “low-chance” results returned?	no return of low-chance results	base	–	–	–	12.8%	3
	online portal	0.09084	0.06886	0.00082	0.818	–	–
	telehealth or phone	0.08464	0.07802	0.00883	0.742	–	–
	in person	0.16241^b^	0.07435	0.35896^b^	0.140	–	–
What happens if new relevant information becomes available?	no updates will be provided	base	–	–	–	9.0%	8
	updates on request	0.11471	0.08046	0.41969^b^	0.193	–	–
	provided automatically in secure online portal	0.10296	0.08113	0.33518	0.207	–	–
gNBS constant	gNBS	4.03379^a^	0.2824	1.20584	1.156	–	–
Log likelihood function	−6716.34419	–	–	–	–	–	–
McFadden pseudo *R*-squared	0.2396169	–	–	–	–	–	–
Akaike information criterion	13514.7	–	–	–	–	–	–

SE, standard error; SD, standard deviation.

Significant SD indicates heterogeneity in preferences.

a Statistically significant at 1% level.

b Statistically significant at 5% level.

Some attributes exhibited significant preference heterogeneity (evidenced by significant standard deviations). These included: gNBS being discussed in the second trimester rather than at the first doctor appointment or later during pregnancy or shortly after birth; that the return of high-chance results should be delivered by a genetics health professional; and whether low-chance results should be returned in person. Having high-chance results returned by genetic health professionals was associated with lower education levels (Table S5). The ranking of importance scores in Table 4 revealed that the most important attribute was “how high-chance results are returned” (24%), followed by “who provides initial information about gNBS screening” (13.2%), “how low-chance results are returned” (12.8%), and “who returns high-chance results” (12.5%).

## Discussion

This study elicited preferences, values, and priorities for gNBS and its implementation from 2,509 members of the Australian public and provides empirical evidence to support the evaluation and potential implementation of gNBS in Australia. Most respondents (72%) indicated preference for only including conditions with onset in infancy in gNBS, with about 50% indicating preference for inclusion of conditions with childhood onset (<5 years of age). Our DCE findings demonstrated that the Australian public has a positive preference for gNBS, with the cost of testing being the most important driver of people’s decision about uptake of gNBS. The expected uptake of gNBS in Australia was estimated to be over 87%. Our results provided insights from the perspective of the Australian public about the inclusion of conditions in gNBS in terms of severity, certainty of developing (penetrance), treatment availability, and diagnoses enabled, highlighting a trade-off between the utility of enabling more diagnoses and the disutility of having less effective (or no) treatments available and greater uncertainty about penetrance. A gNBS program yielding 10–50 additional diagnoses per 1,000 newborns screened relative to standard NBS is valued by the Australian public at AU$4,600–5,700 (US$2,990–3,700) per newborn screened. The overall positive preference for gNBS aligns with the results of other surveys of the public,^27^^,^^28^^,^^29^^,^^30^ qualitative work involving members of the public,^21^^,^^22^^,^^31^ and formal public dialogs.^32^ The high value of genomic sequencing has been further demonstrated across diagnostic applications in pediatric and adult-onset conditions,^16^^,^^33^^,^^34^^,^^35^ and for children with rare genetic diseases,^36^ including complex pediatric neurological disorders,^37^ severe childhood speech disorders,^38^ and critically ill infants and children.^39^ The high value of gNBS may therefore be attributed to the high priority of healthcare interventions for severe and rare conditions,^40^^,^^41^ particularly those affecting children, in the public’s preferences.^42^^,^^43^

Our analysis exploring heterogeneity of preferences indicated that younger people, people familiar with genetic conditions and genomics, and people who are less risk averse were significantly more likely to take up gNBS. These results align with findings from other DCEs^37^ and provide valuable policy insights to support wider and more equitable adoption of gNBS.

The implementation of gNBS warrants careful consideration of service delivery models,^3^^,^^7^^,^^8^^,^^44^ and our results indicated that participants preferred to have the initial communication and return of results from trusted health professionals. While midwives currently provide information and support to prospective parents about standard NBS,^45^ our participants preferred primary care physicians (GPs) or obstetricians for general information and genetic professionals for more complex gNBS results. Primary care physicians currently express hesitancy and indicate that they would require additional training before discussing gNBS,^46^ and upskilling this workforce in gNBS principles, alongside midwives and obstetricians, will be critical. In-person interactions were preferred for delivering both high- and low-chance results. A DCE study by Goranitis et al.^47^ on the process of returning additional findings from genomic testing found that families affected by rare diseases have a preference for an in-person return of high-chance results only. This provides a more feasible way of delivering gNBS at scale, and decisions regarding service delivery models require careful ongoing consideration to balance costs, feasibility, impacts on the healthcare system, and public preferences.

Digital tools will be important to facilitate information delivery and decision support for a gNBS program at scale^48^ and are being trialed.^49^^,^^50^^,^^51^ It has been suggested that the use of decision support tools may encourage greater engagement with the content^7^ and may enable more thorough consideration of gNBS.^52^^,^^53^ The results of our DCE indicate that participants did not have a significant preference for the type of available support material. Parents of children experiencing rare disease have shown strong preference for high-quality online resources and for receiving automatic updates through a secure online portal if new information becomes available,^47^ and perhaps the difference with our findings may be attributed to differences in experiences between members of the public who participated in the study and families affected by a rare disease.

Whether consent should be required for standard NBS or if participation should be mandatory is a debatable topic,^54^ and consent procedures vary across and within countries,^2^ with models ranging from opt-in, where parents must actively agree to participation, to implied consent, where participation is assumed unless parents explicitly decline. In Australia, consent requirements for standard NBS vary depending on jurisdiction, with some states following a written consent model, where parents are provided with information about the screening program and must sign a consent form at the time of blood sample collection, while others operate on an implied consent model.^45^ There is agreement that consent for gNBS should be explicitly provided.^7^ Our results show that the majority of participants preferred an explicit opt-in method of consent (65%).

A number of limitations should be noted. Participants were recruited through an established online panel company. Despite careful recruitment of respondents, unobservable factors related to their participation in this type of research may introduce potential biases. However, this method is widely used and has been shown to be valid and reliable.^55^^,^^56^

Second, stated preference methods are hypothetical in nature. There may be a hypothetical bias introduced if description-based choices are different from experience-based choices. Participants in our study were asked to imagine they had a newborn child and to choose which program they would prefer for this hypothetical child. Across the two surveys, approximately 62% of respondents had children. While having a child was not associated with a higher gNBS uptake rate or systematic preference heterogeneity across DCE attributes, we cannot know how participants would make a choice in real life as gNBS is not yet implemented. While it has been demonstrated that DCEs can produce reasonable predictions of health-related behaviors,^57^ more research is warranted on the external validity of DCEs.

Third, DCEs can only assess preferences for attributes included in the design. This means that preferences for other attributes that were not included could not be estimated. For example, we were interested in including an attribute around the availability of reproductive carrier screening^58^ before pregnancy or early in pregnancy to investigate how such a screening program might affect the uptake of gNBS. However, after piloting we had to exclude the attribute, as it increased complexity for participants to understand how it may interact with gNBS. Other potential attributes such as the consent process for gNBS and the age of onset for conditions were excluded from the DCE but included separately in the survey to reduce the complexity of choice tasks. Exploring how public preferences for gNBS and its implementation vary across jurisdictions would be an important area for further research.

While our sampling approach ensured representation in terms of age, gender, income, and geographical location, we acknowledge that race, ethnicity, and urban status were not included as sampling quotas. Additionally, data on race and ethnicity were not collected, which prevents us from examining potential differences in preferences across diverse populations. Given the importance of ensuring equity of access to genomic newborn screening, future research should explore whether preferences differ by racial or ethnic background, particularly in the context of out-of-pocket costs and systemic barriers to access. Understanding and promoting diversity and inclusion in gNBS and precision medicine is vital to preventing the unintended exacerbation of existing inequities.^59^^,^^60^^,^^61^^,^^62^^,^^63^

As healthcare systems globally explore the integration of genomic sequencing into NBS programs, our study provides critical insights about the preferences, values, and priorities of the Australian public for gNBS and its implementation. Participants prefer broader inclusion criteria for conditions included in gNBS and value accuracy as well as number of diagnoses made through gNBS. We estimate that uptake of gNBS would be over 87% depending on how the program is implemented and the additional diagnoses that are expected to be made. Participants prefer to receive initial information about gNBS from their primary care physician or obstetrician. They prefer to receive high-chance results from a genetics health professional in person, although telehealth and phone communication are also acceptable. For low-chance results, communication in person is favored over other methods. Ensuring that gNBS programs meet public expectations while providing value for money is an important consideration for a high-value public health program delivered at scale. The findings of this work should be considered in the economic evaluation and translation of gNBS in Australia and should inform gNBS implementation efforts in other healthcare systems.

## Data and code availability

The code used for the analysis is available on request, and choice data are available upon request with the limitation that availability of individual-level data is subject to consent/privacy policies.

## Acknowledgments

This work was supported by the Australian Government through the Medical Research Future Fund, as part of the Genomics Health Futures Mission (grant number MRF2015937).

## Author contributions

Conceptualization, all authors; formal analysis, R.P. and I.G.; funding acquisition, S.L., Z.S., and I.G.; methodology, all authors; supervision, Z.S. and I.G., writing – original draft, R.P., Z.S., and I.G., writing – review and editing, all authors.

## Declaration of interests

The authors declare no conflicts of interest.

## Supplemental information

Document S1. Tables S1–S5, supplemental methods, and final surveys

Document S2. Article plus supplemental information
