# Supplementary material for: Public preferences for the value and implementation of genomic newborn screening: Insights from two discrete choice experiments in Australia
Source: Am J Hum Genet. 2025 May 28;112(7):1515–27. doi: 10.1016/j.ajhg.2025.05.001 (PMC12256898; doi:10.1016/j.ajhg.2025.05.001)
Supplement: Document S2. Article plus supplemental information [file mmc2.pdf]

Public preferences for the value and implementation of genomic newborn screening: Insights from two discrete choice experiments in Australia

Authors

Riccarda Peters, Stephanie Best, Fiona Lynch, ..., Sebastian Lunke, Zornitza Stark, Ilias Goranitis

Correspondence

zornitza.stark@vcgs.org.au (Z.S.), ilias.goranitis@unimelb.edu.au (I.G.)

Genomics will potentially transform newborn screening programs globally. To inform implementation, we surveyed 2,509 members of the Australian public to understand the importance they place on key aspects of genomic newborn screening and their preferences about how programs should be delivered in practice.

Peters et al., 2025, The American Journal of Human Genetics *112*, 1515–1527
July 3, 2025 © 2025 The Authors. Published by Elsevier Inc. on behalf of American Society of Human Genetics.
https://doi.org/10.1016/j.ajhg.2025.05.001

ARTICLE

Public preferences for the value and implementation of genomic newborn screening: Insights from two discrete choice experiments in Australia

Riccarda Peters,[1] Stephanie Best,[2,3] Fiona Lynch,[4,5] Danya F. Vears,[4] Lilian Downie,[6,7] Alison D. Archibald,[2,6,7,8] Sebastian Lunke,[7,9] Zornitza Stark,[2,6,7,*] and Ilias Goranitis[1,2,*]

Summary

Integrating genomic sequencing into newborn screening (NBS) has transformative potential for the identification and management of genetic conditions. Using discrete choice experiment surveys, we elicited the preferences, values, and priorities of 2,509 members of the Australian public about the value (n = 1,504) and implementation (n = 1,005) of genomic NBS (gNBS). The Australian public demonstrated positive preference for gNBS, with 90% of respondents indicating an interest in gNBS results. Cost of screening was the most important attribute in people's decision about uptake of gNBS. Enabling diagnosis in more newborns increases the utility of gNBS. To enable these diagnoses, the public is willing to accept less restrictive models of gNBS in terms of the types of conditions included. However, there is disutility associated with including conditions that have less effective (or no) treatments available and including conditions with reduced penetrance. A gNBS program yielding 10–50 additional diagnoses per 1,000 newborns screened relative to standard NBS was valued by the Australian public at AU$4,600–$5,700 (US$2,990–$3,700) per newborn screened. Most participants (65%) preferred an opt-in type of consent and expressed a preference to receive high-chance results in person from a genetics professional, although telehealth and phone options were acceptable. Our findings should inform economic evaluation and future implementation for gNBS in the Australian and other healthcare systems.

Introduction

Newborn screening (NBS) is a public health success story.[1] NBS programs reduce morbidity and mortality through the early identification and management of serious but treatable conditions that benefit from early intervention.[2] NBS programs are implemented in many countries and achieve near-universal uptake.[2] Traditional NBS programs primarily measure biochemical markers in blood collected on blood spot cards within the first 48 h of life using mass spectrometry. While this method is highly accurate, it limits detection to conditions where a biochemical marker is available. New technologies can be used in NBS to look for many more health conditions. One of these technologies is genomic sequencing, which has the potential to revolutionize NBS programs by analyzing hundreds of genes associated with rare diseases simultaneously.[3] Though individually rare, these conditions collectively represent a substantial health and economic burden on the population and health systems.[4] Currently there are many large-scale cohort studies under way internationally generating evidence to guide the implementation of genomic sequencing into NBS programs, commonly referred to as genomic newborn screening (gNBS).[3]

While gNBS offers many opportunities, such as earlier and faster identification of a much wider range of conditions, there are also considerable challenges that need to be addressed if it were implemented as a population-level screening program.[5,6] Importantly, identifying pathogenic variants through genomic screening does not equate to a clinical diagnosis,[3] and robust evidence is needed regarding its clinical utility, accuracy, and impact on health outcomes.[5,6] There are also ethical, legal, and social considerations of gNBS including balancing the best interests of the child and family, selecting appropriate genes, the possibility of secondary or incidental findings, and ensuring equitable access and outcomes.[7] The possible implementation of gNBS at scale requires careful consideration to address both the ethical and practical aspects,[5,6,8] and care must also be taken to maintain high uptake.[9]

Existing NBS programs already take variable approaches to the number of conditions screened and how the service is delivered, such as the amount of information provided to parents or the need for explicit consent.[2] The generation of genomic data will likely amplify these issues.[3,5] Notable variations can be observed within the gNBS pilot studies currently in progress, such as decisions on which conditions to include,[10–13] placing variable emphasis on

[1]Economics of Genomics and Precision Medicine Unit, Centre for Health Policy, Melbourne School of Population and Global Health, The University of Melbourne, Melbourne, VIC 3052, Australia; [2]Australian Genomics, Melbourne, VIC 3052, Australia; [3]Melbourne School of Health Sciences, Faculty of Medicine, Dentistry and Health Sciences, The University of Melbourne, Melbourne, VIC 3052, Australia; [4]Biomedical Ethics Research Group, Murdoch Children's Research Institute, Parkville, VIC, Australia; [5]Melbourne Law School, The University of Melbourne, Melbourne, VIC 3052, Australia; [6]Department of Paediatrics, Faculty of Medicine, Dentistry and Health Sciences, The University of Melbourne, Melbourne, VIC 3052, Australia; [7]Victorian Clinical Genetics Services, Murdoch Children's Research Institute, Parkville, VIC 3052, Australia; [8]Genomics in Society, Murdoch Children's Research Institute, Parkville, VIC, Australia; [9]Department of Clinical Pathology, Faculty of Medicine, Dentistry and Health Sciences, The University of Melbourne, Melbourne, VIC 3052, Australia
*Correspondence: zornitza.stark@vcgs.org.au (Z.S.), ilias.goranitis@unimelb.edu.au (I.G.)
https://doi.org/10.1016/j.ajhg.2025.05.001.
© 2025 The Authors. Published by Elsevier Inc. on behalf of American Society of Human Genetics.
This is an open access article under the CC BY license (http://creativecommons.org/licenses/by/4.0/).

analytical and clinical validity, penetrance, age at condition onset, and actionability.[3] However, harmonization efforts are emerging.[13] From an implementation perspective, to incorporate genomic technologies in NBS, the whole screening pathway will need to be modified, including changes from how information is delivered to parents to support informed decision-making to how data are stored.[3] An important component of ensuring successful and value-based implementation of gNBS is understanding public preferences, values, and priorities.

Discrete choice experiments (DCEs) represent a survey-based method widely used to determine individual preferences for health, healthcare services, technologies, and other goods and services influencing health.[14] DCEs are a valuable tool for eliciting preferences and quantifying how individuals and the public trade off various attributes of health interventions.[15] There is an increasing application of DCEs in the context of genomics,[16,17] and preferences have been used to inform economic evaluations in this context.[17–24]

This study reports on two DCEs conducted as part of the BabyScreen+ program to elicit the Australian public's preferences, values, and priorities for gNBS and its implementation. The first DCE aimed to assess the value of gNBS (value DCE), while the second examined preferences regarding service delivery (implementation DCE), including when and how information about gNBS should be delivered and how results should be returned. The findings provide critical insights into the Australian public preferences and values for gNBS, and its translation and will enable cost-benefit evaluations to support the sustainable implementation of genomics into health systems.

Material and methods

Study design and participants

A DCE survey is a research method used to understand patient or healthcare provider preferences by presenting them with a series of hypothetical scenarios. In each scenario, respondents are asked to choose between different options, each characterized by a set of features or "attributes" (e.g., treatment effectiveness and cost). These attributes have different "levels" (e.g., high vs. low effectiveness). By analyzing the choices people make, DCE surveys help to identify the relative importance of each attribute and the trade-offs respondents are willing to make between them.[18]

The DCE surveys were designed following best practice recommendations for development,[19] analysis,[18] and reporting.[14] As recommended,[20] focus groups were conducted to identify and develop the DCE attributes, and detailed information about the focus groups' methods and findings is available elsewhere.[21,22] Attribute levels were identified in consultation with the genetic experts of the research team to ensure clinical face validity. The final attributes and corresponding levels and descriptions of both DCEs are shown in Tables 1 and 2.

To ensure participants were adequately prepared with information to complete the surveys, the surveys included an educational component, which comprised a video developed by the BabyScreen+ study team to explain gNBS. Participants were further presented with an explanation of gNBS characteristics and were guided through an explanation of the DCE with an example. Participants were then presented with a choice scenario involving two gNBS alternatives and an opt-out option representing the standard NBS, as shown in Figures 1 and 2, respectively. Choice scenarios were designed using a labeled D-efficient partial profile design in Ngene (ChoiceMetrics [2024] Ngene 1.4 User Manual and Reference Guide, Australia, https://www.choice-metrics.com/NgeneManual140.pdf), which helps reduce task complexity. The design for the value DCE included 32 choice tasks split across four blocks, while 64 choice tasks were split across six blocks for the implementation DCE. Thus, for both surveys each participant was required to complete eight choice tasks. Full details about the experimental design used in the surveys are provided in the supplemental methods.

The surveys were developed with support from a plain-language advisor and piloted using think-aloud interviews among a convenience sample of university staff to assess completion time, language, and understanding ($n = 5$). The surveys underwent several rounds of piloting ($n = 873$) to evaluate whether the coefficients were logically ordered, to assess completion time, and to gather feedback on the difficulty of the task. The pilot results were used to refine the DCE experimental design and final survey wording. The final surveys are available in the supplemental information.

In accordance with recommended practices on the measurement and valuation of health benefits for economic evaluation,[23] we sought the values of the general public, who are both taxpayers and potential users of healthcare. Two independent Australia-wide samples of participants over the age of 18 years were recruited from nationwide panels through the research market company Pureprofile. Age, gender, income, and geographical location (states) quotas were used to ensure that the sample was representative of the Australian public. This was further validated against other national sources.[24]

Ethics statement

Informed consent was obtained from all participants before entering the survey. Ethics approval was granted from the Royal Children's Hospital Melbourne Human Research Ethics Committee (Ethics ID: HREC/91392/RCHM-2022).

Choice analysis

Choice data were analyzed using a panel error component mixed logit model, which uses random parameters to account for unobserved heterogeneity of preferences among participants.[18] Full information about the coding of the attributes and analytical methods used in choice analysis is provided in the supplemental methods. We estimate the relative importance of each attribute based on the proportional change in overall utility associated with transitioning from the lowest to the highest level of each attribute.[25] We further explore preference heterogeneity across both DCEs using a variety of methods including a latent class choice model, which divides the sample into a finite number of groups (classes) with homogeneous preferences. Full information on the econometric analysis of our choice data is available in the supplemental methods. The results of the latent class model were used to provide an estimate of the uptake of gNBS based on the proportion of participants demonstrating no preference for gNBS and its attributes.

Finally, we estimate the overall value that the public attaches to a publicly funded gNBS with a restrictive and non-restrictive

Table 1. Attributes and attribute levels included in the value discrete choice experiment (DCE)

Attributes	Definition	Levels
Severity of conditions (without treatment)	how severe the conditions included in the genomic newborn screening program are in the absence of treatment. Severity was defined based on impact on quality of life and life expectancy, ranging from minimal to significant effects on both dimensions	1. Profound only
		2. Profound and moderate
		3. Profound, moderate, and mild
Certainty that the condition develops (without treatment)	information about how likely it is that the condition develops. If genomic newborn screening shows a high chance that a child might develop a condition, it is not certain the child will end up developing the condition. For example: high certainty means that medical experts are very confident (90%) that the condition will develop	1. High, moderate, and average certainty (greater than 50%)
		2. High and moderate certainty (greater than 75%)
		3. High certainty only (greater than 90%)
Treatment availability for conditions screened	indicates whether there are ways to treat the conditions diagnosed from genetic newborn screening. Some conditions can be treated to either cure them or manage their symptoms. For others, there are no treatments available at the moment	1. Treatments that cure conditions
		2. Treatments that cure conditions or manage their symptoms
		3. Treatments that cure conditions or manage their symptoms and conditions without current treatment
Additional number of newborns diagnosed compared to standard newborn screening (in every 1,000 newborns screened)	additional number of newborns that will be identified with a high chance of developing a condition through genomic newborn screening compared to standard newborn screening	1. 3 in 1,000
		2. 10 in 1,000
		3. 20 in 1,000
		4. 50 in 1,000
Accuracy of screening results	how likely it is that the screening results are correct. Capturing the overall reliability of the screening results	1. 95% (5 out of 100 screened newborns will receive a wrong initial diagnosis)
		2. 98% (2 out of 100 screened newborns will receive a wrong initial diagnosis)
		3. 100% (none of the newborns will receive a wrong initial diagnosis)
Cost of genomic newborn screening to you	because genomic newborn screening is a new program, the Federal Government may not pay for it. This characteristic tells you how much you would need to pay out-of-pocket for genomic newborn screening	1. $500
		2. $1,000
		3. $2,500

gNBS implementation relative to standard NBS using the compensating variation method.[26] The restrictive gNBS model includes conditions of profound severity, high chance of developing (>90%), and curative treatments available. The non-restrictive gNBS model includes conditions of profound, moderate, and mild severity, conditions with >50% chance of developing, conditions with curative treatments available, and treatments to manage symptoms and conditions without current treatment. The values are reported in both Australian and US dollars (using November 21, 2024 Reserve Bank of Australia exchange rate of 0.65). Analyses were performed in Nlogit 6 (Econometric Software, Waverton, NSW, Australia) and Stata (StataCorp, College Station, TX, USA).

Results

Demographics

Overall, 2,509 members of the Australian public participated in the value (n = 1,504) and implementation (n = 1,005) DCEs. The samples were similar in terms of age, gender, household income, and geographical location compared to the national census summary.[24] Detailed information about the socioeconomic status, demographics, and responses regarding experience with genomics and knowledge about genetic conditions and newborn screening is provided in Table S1.

Across the two surveys, 16% of respondents had experience with a genetic condition, 20% had experience with genetic or genomic testing, 42% had heard about genomic testing before the survey, and about half of respondents knew a little (43%) to a lot (6%) about newborn screening before participating.

A total of 1,504 participants were asked to indicate their preference for which conditions should be included in gNBS based on age of onset of the condition. Multiple selections were allowed. The majority indicated a preference for conditions with an onset in infancy (72%) and early childhood (49%) (Table S2). About 30% of respondents

Table 2. Attributes and attribute levels included in the implementation DCE

Attributes	Levels
When is genomic newborn screening first discussed?	early during pregnancy at first doctor appointment
	second trimester
	third trimester
	shortly after birth
Who provides initial information?	a midwife or nurse
	your GP
	an obstetrician
	a genetic health professional
What support material is available?	no support material
	leaflet
	interactive online portal
	appointment with health professional
What conditions are included?	conditions selected by parents
	conditions selected by health professionals
Who returns "high-chance" results?	your GP
	a relevant medical specialist
	a genetic health professional
How are "high-chance" results returned?	electronically through a secure online portal
	directly, telehealth, or phone
	directly, in person
How are "low-chance" results returned?	no return of "low-chance" results
	electronically through a secure online portal
	directly, telehealth, or phone
	directly, in person
What happens if new relevant information becomes available?	no updates will be provided
	updates will be provided upon request
	updates will be provided automatically in a secure online portal

indicated preference for conditions with onset in childhood and adolescence and 23% for adult-onset conditions, with 10% of respondents indicating no preference for gNBS. Another group of 1,005 participants were asked to indicate their preferred method of consent for gNBS. The majority of participants (65%) preferred an opt-in consent process (Table S2).

Value DCE results and interpretation

The results of the Value DCE are presented in Table 3. The members of the public demonstrated statistically significant preferences across all gNBS attributes. Cost of testing was the most significant driver in people's choice to have gNBS or not. Accuracy in screening results also increases the utility for gNBS. Enabling diagnosis in more newborns was an important driver of respondents' utility for gNBS, and, as such, respondents on average were willing to accept gNBS models that included conditions of moderate and mild severity, conditions with moderate and average certainty of developing, and conditions with treatments to manage symptoms as well as conditions without current treatment available. However, when controlling for the number of diagnoses achieved through restrictive and less restrictive gNBS models, it is evident that respondents preferred more restrictive gNBS models (i.e., inclusion of conditions with profound severity only, high certainty of developing, and curative treatments available). This means that as more diagnoses are enabled through gNBS, the value of more conservative gNBS models is increasing, while the value of less restrictive models remains relatively stable given that the added utility from new diagnoses is mitigated by the disutility of having less effective (or no) treatments available and greater uncertainty about whether a condition will develop. As shown in Figure 3, the value of a restrictive gNBS model increases from AU$4,600 (US$2,990) to $5,700 (US $3,700) per newborn screened as the incremental screening yield of gNBS relative to standard NBS increases from 10 to 50 diagnoses per 1,000 newborns screened, whereas the value of a non-restrictive gNBS model remains relatively stable at AU$5,400 (US$3,510) per newborn screened.

The standard deviations for most parameters were statistically significant, indicating heterogeneity of preferences among respondents (Table 3). The latent class analysis revealed four groups (classes) of participants with more homogeneous preferences (Table S3). Class 1 corresponds to people who had a negative preference for gNBS, evidenced by the high negative constant, and made up 13% of the overall sample. However, the remaining 87% had a positive preference for gNBS. Class 2 comprises 28% of the sample and includes people with positive preference for testing who demonstrated disutility for higher costs and utility for screening accuracy. Classes 3 and 4, comprising 42% and 17% of the sample, were distinguished based on how they valued different attributes: cost and number of diagnoses were not significant drivers of choice-making for class 3 but were major choice determinants in class 4. Class 1 participants tended to be of higher age, lower education, and lower income. They also tended to have had children, had less experience with genetic conditions, and had less knowledge about genetic conditions (Table S4). Class 3 participants tended not to have children, had experience with and had knowledge about genetic conditions, and had higher health literacy.

Results of implementation DCE

The results of the implementation DCE are shown in Table 4. Participants exhibited greater utility for receiving initial information about the screening program from their general practitioner (GP) or obstetrician. Participants demonstrated preference for receiving results that indicate a high chance for their child having a genetic

	Genomic Newborn Screening 1	Genomic Newborn Screening 2	Standard Newborn Screening only
**Severity of the condition**	Profound moderate & mild	Profound & moderate	
**Certainty that the condition develops**	High moderate & average certainty (greater than 50%)	High moderate & average certainty (greater than 50%)	
**Availability of treatment**	Treatments that cure conditions or manage their symptoms	Treatments that cure conditions or manage their symptoms	
**Additional number of newborns diagnosed compared to standard newborn screening (in every 1000 newborns screened)**	50 in 1000	20 in 1000	
**Accuracy of screening results**	100% (none of the newborns screened will receive a wrong initial diagnosis)	95% (5 out of 100 newborns screened will receive a wrong initial diagnosis)	
**Cost of genomic newborn screening to you**	$2,500	$1,000	$0

**I would prefer:**	○ Genomic Newborn Screening 1	○ Genomic Newborn Screening 2	○ Standard Newborn Screening only

Figure 1. Choice task example for the value DCE

condition (high-chance result) from a genetics health professional either in person or through telehealth or phone. Even low-chance results, when screening does not detect any increased chance of developing the medical problems screened for, were preferred to be communicated in person rather than electronically.

Some attributes exhibited significant preference heterogeneity (evidenced by significant standard deviations). These included: gNBS being discussed in the second trimester rather than at the first doctor appointment or later during pregnancy or shortly after birth; that the return of high-chance results should be delivered by a genetics health professional; and whether low-chance results should be returned in person. Having high-chance results returned by genetic health professionals was associated with lower education levels (Table S5). The ranking of importance scores in Table 4 revealed that the most important attribute was "how high-chance results are returned" (24%), followed by "who provides initial information about gNBS screening" (13.2%), "how low-chance results are returned" (12.8%), and "who returns high-chance results" (12.5%).

Discussion

This study elicited preferences, values, and priorities for gNBS and its implementation from 2,509 members of the Australian public and provides empirical evidence to support the evaluation and potential implementation of gNBS in Australia. Most respondents (72%) indicated preference for only including conditions with onset in infancy in gNBS, with about 50% indicating preference for inclusion of conditions with childhood onset (<5 years of age). Our DCE findings demonstrated that the Australian public has a positive preference for gNBS, with the cost of testing being the most important driver of people's decision about uptake of gNBS. The expected uptake of gNBS in Australia was estimated to be over 87%. Our results provided insights from the perspective of the

	Genomic Newborn Screening 1	Genomic Newborn Screening 2
**When** is genomic newborn screening first discussed?	Early during pregnancy at first doctor appointment	Shortly after birth
**Who** returns "high chance" results?	Your GP	A genetic health professional
**How** are "high chance" results returned?	Electronically through a secure online portal	Directly, in person
What happens if **new relevant information** becomes available?	Updates will be provided upon request	No updates will be provided

**I would choose:**	☐ **Genomic Newborn Screening 1**	☐ **Genomic Newborn Screening 2**	☐ **Neither**

Figure 2. Choice task example for the implementation DCE

Table 3. Attribute marginal utilities and importance scores for the value DCE

	Attributes	Mean	SE	SD	SE	Importance score (%)	Ranking
Severity	profound conditions only	base	–	–	–	16.4%	2
	profound and moderate conditions	0.85198[a]	0.1537	0.57285	0.3419	–	–
	profound, moderate, and mild conditions	1.35147[a]	0.1372	1.44105[a]	0.13944	–	–
Certainty	high, moderate, and average certainty (greater than 50%)	base	–	–	–	8.9%	6
	high and moderate certainty (greater than 75%)	−0.36018[a]	0.1671	0.94002[a]	0.3009	–	–
	high certainty only (greater than 90%)	−0.73117[a]	0.1103	0.62383[a]	0.1597	–	–
Treatment availability	treatments that cure conditions	base	–	–	–	11.2%	5
	treatments that cure conditions or manage their symptoms	0.51008[a]	0.1221	0.00518	0.4171	–	–
	treatments that cure conditions or manage their symptoms and conditions without current treatment	0.92122[a]	0.1216	1.20558[a]	0.12109	–	–
Additional number of newborns diagnosed compared to standard newborn screening (in every 1,000 newborns screened)	–	0.0266[a]	0.0060	0.0635[a]	0.0031	15.2%	4
Accuracy of screening results	95%	base	–	–	–	16.2%	3
	98%	0.83427[a]	0.0644	0.31191	0.17916	–	–
	100%	1.331[a]	0.0668	1.25738[a]	0.0765	–	–
Cost of testing to you (AU$)	–	−0.00132[a]	0.5301D-04	0.00132[a]	0.5301D-04	32.1%	1
Genomic newborn screening constant	–	4.97359[a]	0.3318	6.36818[a]	0.3192	–	–
**Interactions**							
Additional diagnoses × profound and moderate conditions	–	−0.01599[a]	0.0047	0.00319	0.123	–	–
Additional diagnoses × profound, moderate, and mild conditions	–	−0.01403[a]	0.0054	0.3187[a]	0.0054	–	–
Additional diagnoses × high and moderate certainty (greater than 75%)	–	−0.01029[a]	0.0046	0.00669	0.01028	–	–
Additional diagnoses × high certainty only (greater than 90%)	–	0.01744[a]	0.0051	0.1209	0.0090	–	–
Additional diagnoses × treatments that cure conditions or manage their symptoms	–	−0.00673	0.0050	0.01661[a]	0.00683	–	–

(*Continued on next page*)

Table 3. Continued

	Attributes	Mean	SE	SD	SE	Importance score (%)	Ranking
Additional diagnoses × treatments that cure conditions or manage their symptoms and conditions without current treatment	–	−0.01735[a]	0.0047	0.00713	0.0078	–	–
Log likelihood function	−8183.22114	–	–	–	–	–	–
McFadden pseudo R-squared	0.3809268	–	–	–	–	–	–
Akaike information criterion	16434.4	–	–	–	–	–	–

SE, standard error; SD, standard deviation.
Regression coefficients show the marginal impact of each attribute (or attribute level) on the utility of genomic newborn screening. Positive (or negative) mean estimates suggest an average positive (or negative) effect on utility. SD estimates illustrate the variability in preferences among the study participants.
[a]Statistically significant at 1% level.

Australian public about the inclusion of conditions in gNBS in terms of severity, certainty of developing (penetrance), treatment availability, and diagnoses enabled, highlighting a trade-off between the utility of enabling more diagnoses and the disutility of having less effective (or no) treatments available and greater uncertainty about penetrance. A gNBS program yielding 10–50 additional diagnoses per 1,000 newborns screened relative to standard NBS is valued by the Australian public at AU$4,600–5,700 (US$2,990–3,700) per newborn screened. The overall positive preference for gNBS aligns with the results of other surveys of the public,[27–30] qualitative work involving members of the public,[21,22,31] and formal public dialogs.[32] The high value of genomic sequencing has been further demonstrated across diagnostic applications in pediatric and adult-onset conditions,[16,33–35] and for children with rare genetic diseases,[36] including complex pediatric neurological disorders,[37] severe childhood speech disorders,[38] and critically ill infants and children.[39] The high value of gNBS may therefore be attributed to the high priority of healthcare interventions for severe and rare conditions,[40,41] particularly those affecting children, in the public's preferences.[42,43]

Our analysis exploring heterogeneity of preferences indicated that younger people, people familiar with genetic conditions and genomics, and people who are less risk averse were significantly more likely to take up gNBS. These results align with findings from other DCEs[37] and provide valuable policy insights to support wider and more equitable adoption of gNBS.

The implementation of gNBS warrants careful consideration of service delivery models,[3,7,8,44] and our results indicated that participants preferred to have the initial communication and return of results from trusted health professionals. While midwives currently provide information and support to prospective parents about standard NBS,[45] our participants preferred primary care physicians (GPs) or obstetricians for general information and genetic professionals for more complex gNBS results. Primary care physicians currently express hesitancy and indicate that they would require additional training before discussing gNBS,[46] and upskilling this workforce in gNBS principles, alongside midwives and obstetricians, will be critical. In-person interactions were preferred for delivering both high- and low-chance results. A DCE study by Goranitis et al.[47] on the process of returning additional findings from genomic testing found that families affected by rare diseases have a preference for an in-person return of high-chance results only. This provides a more feasible way of delivering gNBS at scale, and decisions regarding service delivery models require careful ongoing consideration to balance costs, feasibility, impacts on the healthcare system, and public preferences.

Digital tools will be important to facilitate information delivery and decision support for a gNBS program at scale[48] and are being trialed.[49–51] It has been suggested

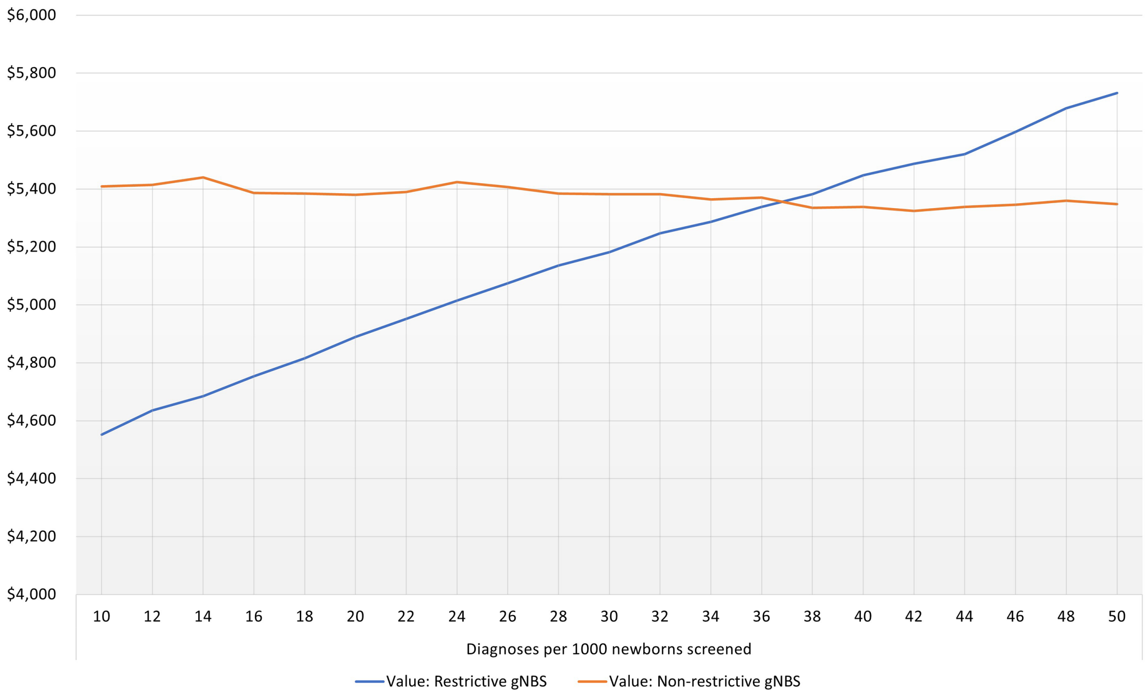

Figure 3. The incremental value in Australian dollars of publicly funded genomic newborn screening (gNBS) applications relative to standard NBS as a function of the additional diagnoses enabled per 1,000 newborns screened
Restrictive gNBS includes conditions of profound severity, high chance of developing (>90%), and curative treatments available; non-restrictive gNBS includes conditions of profound, moderate, and mild severity, conditions with >50% chance of developing, conditions with curative treatments available and treatments to manage symptoms, and conditions without current treatment.

that the use of decision support tools may encourage greater engagement with the content[7] and may enable more thorough consideration of gNBS.[52,53] The results of our DCE indicate that participants did not have a significant preference for the type of available support material. Parents of children experiencing rare disease have shown strong preference for high-quality online resources and for receiving automatic updates through a secure online portal if new information becomes available,[47] and perhaps the difference with our findings may be attributed to differences in experiences between members of the public who participated in the study and families affected by a rare disease.

Whether consent should be required for standard NBS or if participation should be mandatory is a debatable topic,[54] and consent procedures vary across and within countries,[2] with models ranging from opt-in, where parents must actively agree to participation, to implied consent, where participation is assumed unless parents explicitly decline. In Australia, consent requirements for standard NBS vary depending on jurisdiction, with some states following a written consent model, where parents are provided with information about the screening program and must sign a consent form at the time of blood sample collection, while others operate on an implied consent model.[45] There is agreement that consent for gNBS should be explicitly provided.[7] Our results show that the majority of participants preferred an explicit opt-in method of consent (65%).

A number of limitations should be noted. Participants were recruited through an established online panel company. Despite careful recruitment of respondents, unobservable factors related to their participation in this type of research may introduce potential biases. However, this method is widely used and has been shown to be valid and reliable.[55,56]

Second, stated preference methods are hypothetical in nature. There may be a hypothetical bias introduced if description-based choices are different from experience-based choices. Participants in our study were asked to imagine they had a newborn child and to choose which program they would prefer for this hypothetical child. Across the two surveys, approximately 62% of respondents had children. While having a child was not associated with a higher gNBS uptake rate or systematic preference heterogeneity across DCE attributes, we cannot know how participants would make a choice in real life as gNBS is not yet implemented. While it has been demonstrated that DCEs can produce reasonable predictions of health-related behaviors,[57] more research is warranted on the external validity of DCEs.

Third, DCEs can only assess preferences for attributes included in the design. This means that preferences for other attributes that were not included could not be estimated. For example, we were interested in including an attribute around the availability of reproductive carrier screening[58] before pregnancy or early in pregnancy to investigate how such a screening program might affect

Table 4. **Attribute marginal utilities, importance scores, and ranking for the implementation DCE**

Attribute	Level	Mean	SE	SD	SE	Importance score (%)	Ranking
When is gNBS first discussed?	first doctor appointment	base	–	–	–	9.5%	6
	second trimester	0.07769	0.0802	0.34563[b]	0.135	–	–
	third trimester	0.11337	0.07463	0.13863	0.334	–	–
	shortly after birth	0.12099	0.07505	0.0129	1.497	–	–
Who provides initial information?	a midwife or nurse	base	–	–	–	13.2%	2
	your GP	0.16861[b]	0.07363	0.06516	0.624	–	–
	an obstetrician	0.14897[b]	0.07194	0.03184	1.071	–	–
	a genetic health professional	0.08439	0.06126	0.19297	0.187	–	–
What support material is available?	no support material	base	–	–	–	9.9%	5
	leaflet	0.12639	0.07	0.06516	1.100	–	–
	interactive online portal	0.09817	0.07619	0.03184	0.212	–	–
	appointment with health professional	0.11875	0.07538	0.19297	0.176	–	–
What conditions are included?	selected by parents	base	–	–	–	9.1%	7
	selected by health professionals	0.11541	0.08652	0.56115[b]	0.237	–	–
Who returns “high-chance” results?	your GP	base	–	–	–	12.5%	4
	a relevant medical specialist	0.12933	0.0799	0.28424	0.241	–	–
	a genetic health professional	0.15933[b]	0.07224	0.42372[a]	0.154	–	–
How are “high-chance” results returned?	online portal	base	–	–	–	24.0%	1
	telehealth or phone	0.26060[a]	0.08248	0.30187	0.225	–	–
	in person	0.30525[a]	0.073	0.32142	0.200	–	–
How are “low-chance” results returned?	no return of low-chance results	base	–	–	–	12.8%	3
	online portal	0.09084	0.06886	0.00082	0.818	–	–
	telehealth or phone	0.08464	0.07802	0.00883	0.742	–	–
	in person	0.16241[b]	0.07435	0.35896[b]	0.140	–	–
What happens if new relevant information becomes available?	no updates will be provided	base	–	–	–	9.0%	8
	updates on request	0.11471	0.08046	0.41969[b]	0.193	–	–
	provided automatically in secure online portal	0.10296	0.08113	0.33518	0.207	–	–
gNBS constant	gNBS	4.03379[a]	0.2824	1.20584	1.156	–	–
Log likelihood function	−6716.34419	–	–	–	–	–	–
McFadden pseudo *R*-squared	0.2396169	–	–	–	–	–	–
Akaike information criterion	13514.7	–	–	–	–	–	–

SE, standard error; SD, standard deviation.
Significant SD indicates heterogeneity in preferences.
[a]Statistically significant at 1% level.
[b]Statistically significant at 5% level.

the uptake of gNBS. However, after piloting we had to exclude the attribute, as it increased complexity for participants to understand how it may interact with gNBS. Other potential attributes such as the consent process for gNBS and the age of onset for conditions were excluded from the DCE but included separately in the survey to reduce the complexity of choice tasks. Exploring how public preferences for gNBS and its implementation vary across jurisdictions would be an important area for further research.

While our sampling approach ensured representation in terms of age, gender, income, and geographical location, we acknowledge that race, ethnicity, and urban status were not included as sampling quotas. Additionally, data on race and ethnicity were not collected, which prevents us from examining potential differences in preferences

across diverse populations. Given the importance of ensuring equity of access to genomic newborn screening, future research should explore whether preferences differ by racial or ethnic background, particularly in the context of out-of-pocket costs and systemic barriers to access. Understanding and promoting diversity and inclusion in gNBS and precision medicine is vital to preventing the unintended exacerbation of existing inequities.[59–63]

As healthcare systems globally explore the integration of genomic sequencing into NBS programs, our study provides critical insights about the preferences, values, and priorities of the Australian public for gNBS and its implementation. Participants prefer broader inclusion criteria for conditions included in gNBS and value accuracy as well as number of diagnoses made through gNBS. We estimate that uptake of gNBS would be over 87% depending on how the program is implemented and the additional diagnoses that are expected to be made. Participants prefer to receive initial information about gNBS from their primary care physician or obstetrician. They prefer to receive high-chance results from a genetics health professional in person, although telehealth and phone communication are also acceptable. For low-chance results, communication in person is favored over other methods. Ensuring that gNBS programs meet public expectations while providing value for money is an important consideration for a high-value public health program delivered at scale. The findings of this work should be considered in the economic evaluation and translation of gNBS in Australia and should inform gNBS implementation efforts in other healthcare systems.

Data and code availability

The code used for the analysis is available on request, and choice data are available upon request with the limitation that availability of individual-level data is subject to consent/privacy policies.

Acknowledgments

This work was supported by the Australian Government through the Medical Research Future Fund, as part of the Genomics Health Futures Mission (grant number MRF2015937).

Author contributions

Conceptualization, all authors; formal analysis, R.P. and I.G.; funding acquisition, S.L., Z.S., and I.G.; methodology, all authors; supervision, Z.S. and I.G., writing – original draft, R.P., Z.S., and I. G., writing – review and editing, all authors.

Declaration of interests

The authors declare no conflicts of interest.

Supplemental information

Supplemental information can be found online at https://doi.org/10.1016/j.ajhg.2025.05.001.

Received: December 18, 2024
Accepted: May 5, 2025
Published: May 28, 2025

References

1. Centers for Disease Control and Prevention (CDC) (2011). Ten Great Public Health Achievements – United States, 2001-2010 (Centers for Disease Control and Prevention (CDC)). https://www.cdc.gov/mmwr/preview/mmwrhtml/mm6019a5.htm.
2. Therrell, B.L., Padilla, C.D., Borrajo, G.J.C., Khneisser, I., Schielen, P.C.J.I., Knight-Madden, J., Malherbe, H.L., and Kase, M. (2024). Current Status of Newborn Bloodspot Screening Worldwide 2024: A Comprehensive Review of Recent Activities (2020–2023). Int. J. Neonatal Screen. *10*, 38. https://doi.org/10.3390/ijns10020038.
3. Stark, Z., and Scott, R.H. (2023). Genomic newborn screening for rare diseases. Nat. Rev. Genet. *24*, 755–766. https://doi.org/10.1038/s41576-023-00621-w.
4. Gonzaludo, N., Belmont, J.W., Gainullin, V.G., and Taft, R.J. (2019). Estimating the burden and economic impact of pediatric genetic disease. Genet. Med. *21*, 1781–1789. https://doi.org/10.1038/s41436-018-0398-5.
5. Baple, E.L., Scott, R.H., Banka, S., Buchanan, J., Fish, L., Wynn, S., Wilkinson, D., Ellard, S., MacArthur, D.G., and Stark, Z. (2024). Exploring the benefits, harms and costs of genomic newborn screening for rare diseases. Nat. Med. *30*, 1823–1825. https://doi.org/10.1038/s41591-024-03055-x.
6. Turnbull, C., Firth, H.V., Wilkie, A.O.M., Newman, W., Raymond, F.L., Tomlinson, I., Lachmann, R., Wright, C.F., Wordsworth, S., George, A., et al. (2024). Population screening requires robust evidence—genomics is no exception. Lancet *403*, 583–586. https://doi.org/10.1016/S0140-6736(23)02295-X.
7. Downie, L., Halliday, J., Lewis, S., and Amor, D.J. (2021). Principles of Genomic Newborn Screening Programs: A Systematic Review. JAMA Netw. Open *4*, e2114336. https://doi.org/10.1001/jamanetworkopen.2021.14336.
8. Vears, D.F., Savulescu, J., Christodoulou, J., Wall, M., and Newson, A.J. (2023). Are We Ready for Whole Population Genomic Sequencing of Asymptomatic Newborns? PGPM *16*, 681–691. https://doi.org/10.2147/PGPM.S376083.
9. Armstrong, B., Christensen, K.D., Genetti, C.A., Parad, R.B., Robinson, J.O., Blout Zawatsky, C.L., Zettler, B., Beggs, A.H., Holm, I.A., Green, R.C., et al. (2022). Parental Attitudes Toward Standard Newborn Screening and Newborn Genomic Sequencing: Findings From the BabySeq Study. Front. Genet. *13*, 867371.
10. Ceyhan-Birsoy, O., Machini, K., Lebo, M.S., Yu, T.W., Agrawal, P.B., Parad, R.B., Holm, I.A., McGuire, A., Green, R.C., Beggs, A.H., and Rehm, H.L. (2017). A curated gene list for reporting results of newborn genomic sequencing. Genet. Med. *19*, 809–818. https://doi.org/10.1038/gim.2016.193.
11. Kingsmore, S.F., Smith, L.D., Kunard, C.M., Bainbridge, M., Batalov, S., Benson, W., Blincow, E., Caylor, S., Chambers, C., Del Angel, G., et al. (2022). A genome sequencing system for universal newborn screening, diagnosis, and precision medicine for severe genetic diseases. Am. J. Hum. Genet. *109*, 1605–1619. https://doi.org/10.1016/j.ajhg.2022.08.003.

12. Milko, L.V., O'Daniel, J.M., DeCristo, D.M., Crowley, S.B., Foreman, A.K.M., Wallace, K.E., Mollison, L.F., Strande, N. T., Girnary, Z.S., Boshe, L.J., et al. (2019). An Age-Based Framework for Evaluating Genome-Scale Sequencing Results in Newborn Screening. J. Pediatr. *209*, 68–76. https://doi.org/10.1016/j.jpeds.2018.12.027.
13. Downie, L., Bouffler, S.E., Amor, D.J., Christodoulou, J., Yeung, A., Horton, A.E., Macciocca, I., Archibald, A.D., Wall, M., Caruana, J., et al. (2024). Gene selection for genomic newborn screening: Moving toward consensus? Genet. Med. *26*, 101077. https://doi.org/10.1016/j.gim.2024.101077.
14. Ride, J., Goranitis, I., Meng, Y., LaBond, C., and Lancsar, E. (2024). A Reporting Checklist for Discrete Choice Experiments in Health: The DIRECT Checklist. Pharmacoeconomics *42*, 1161–1175. https://doi.org/10.1007/s40273-024-01431-6.
15. M. Ryan, K. Gerard, and M. Amaya-Amaya, eds. (2008). Using Discrete Choice Experiments to Value Health and Health Care (Springer Netherlands). https://doi.org/10.1007/978-1-4020-5753-3.
16. Ozdemir, S., Lee, J.J., Chaudhry, I., and Ocampo, R.R.Q. (2022). A Systematic Review of Discrete Choice Experiments and Conjoint Analysis on Genetic Testing. Patient *15*, 39–54. https://doi.org/10.1007/s40271-021-00531-1.
17. Soekhai, V., de Bekker-Grob, E.W., Ellis, A.R., and Vass, C.M. (2019). Discrete Choice Experiments in Health Economics: Past, Present and Future. Pharmacoeconomics *37*, 201–226. https://doi.org/10.1007/s40273-018-0734-2.
18. Hensher, D.A., Rose, J.M., and Greene, W.H. (2015). Applied Choice Analysis, 2nd ed. (Cambridge University Press). https://doi.org/10.1017/CBO9781316136232.
19. Reed Johnson, F., Lancsar, E., Marshall, D., Kilambi, V., Mühlbacher, A., Regier, D.A., Bresnahan, B.W., Kanninen, B., and Bridges, J.F.P. (2013). Constructing Experimental Designs for Discrete-Choice Experiments: Report of the ISPOR Conjoint Analysis Experimental Design Good Research Practices Task Force. Value Health *16*, 3–13. https://doi.org/10.1016/j.jval.2012.08.2223.
20. Coast, J., and Horrocks, S. (2007). Developing attributes and levels for discrete choice experiments using qualitative methods. J. Health Serv. Res. Policy *12*, 25–30. https://doi.org/10.1258/135581907779497602.
21. Lynch, F., Best, S., Gaff, C., Downie, L., Archibald, A.D., Gyngell, C., Goranitis, I., Peters, R., Savulescu, J., Lunke, S., et al. (2024). Australian Public Perspectives on Genomic Newborn Screening: Risks, Benefits, and Preferences for Implementation. Int. J. Neonatal Screen. *10*, 6. https://doi.org/10.3390/ijns10010006.
22. Lynch, F., Best, S., Gaff, C., Downie, L., Archibald, A.D., Gyngell, C., Goranitis, I., Peters, R., Savulescu, J., Lunke, S., et al. (2024). Australian public perspectives on genomic newborn screening: which conditions should be included? Hum. Genomics *18*, 45. https://doi.org/10.1186/s40246-024-00611-x.
23. Brazier, J., Ratcliffe, J., Saloman, J., and Tsuchiya, A. (2016). Measuring and Valuing Health Benefits for Economic Evaluation (Oxford University Press). https://doi.org/10.1093/med/9780198725923.001.0001.
24. Australian Bureau of Statistics (ABS). 2021 Census All persons QuickStats. (2021). https://www.abs.gov.au/census/find-census-data/quickstats/2021/AUS.
25. Gonzalez, J.M. (2019). A Guide to Measuring and Interpreting Attribute Importance. Patient *12*, 287–295. https://doi.org/10.1007/s40271-019-00360-3.
26. Small, K.A., and Rosen, H.S. (1981). Applied Welfare Economics with Discrete Choice Models. Econometrica *49*, 105–130. https://doi.org/10.2307/1911129.
27. Bombard, Y., Miller, F.A., Hayeems, R.Z., Barg, C., Cressman, C., Carroll, J.C., Wilson, B.J., Little, J., Avard, D., Painter-Main, M., et al. (2014). Public views on participating in newborn screening using genome sequencing. Eur. J. Hum. Genet. *22*, 1248–1254. https://doi.org/10.1038/ejhg.2014.22.
28. Goldenberg, A.J., Dodson, D.S., Davis, M.M., and Tarini, B.A. (2014). Parents' interest in whole-genome sequencing of newborns. Genet. Med. *16*, 78–84. https://doi.org/10.1038/gim.2013.76.
29. Waisbren, S.E., Bäck, D.K., Liu, C., Kalia, S.S., Ringer, S.A., Holm, I.A., and Green, R.C. (2015). Parents are interested in newborn genomic testing during the early postpartum period. Genet. Med. *17*, 501–504. https://doi.org/10.1038/gim.2014.139.
30. White, S., Mossfield, T., Fleming, J., Barlow-Stewart, K., Ghedia, S., Dickson, R., Richards, F., Bombard, Y., and Wiley, V. (2023). Expanding the Australian Newborn Blood Spot Screening Program using genomic sequencing: do we want it and are we ready? Eur. J. Hum. Genet. *31*, 703–711. https://doi.org/10.1038/s41431-023-01311-1.
31. Joseph, G., Chen, F., Harris-Wai, J., Puck, J.M., Young, C., and Koenig, B.A. (2016). Parental Views on Expanded Newborn Screening Using Whole-Genome Sequencing. Pediatrics *137*, S36–S46. https://doi.org/10.1542/peds.2015-3731H.
32. Kinsella, S., Hopkins, H., Cooper, L., and Bonham, J.R. (2022). A Public Dialogue to Inform the Use of Wider Genomic Testing When Used as Part of Newborn Screening to Identify Cystic Fibrosis. Int. J. Neonatal Screen. *8*, 32. https://doi.org/10.3390/ijns8020032.
33. Goranitis, I., Best, S., Christodoulou, J., Stark, Z., and Boughtwood, T. (2020). The personal utility and uptake of genomic sequencing in pediatric and adult conditions: eliciting societal preferences with three discrete choice experiments. Genet. Med. *22*, 1311–1319. https://doi.org/10.1038/s41436-020-0809-2.
34. Meng, Y., Clarke, P.M., and Goranitis, I. (2022). The Value of Genomic Testing: A Contingent Valuation Across Six Child- and Adult-Onset Genetic Conditions. Pharmacoeconomics *40*, 215–223. https://doi.org/10.1007/s40273-021-01103-9.
35. Regier, D.A., Weymann, D., Buchanan, J., Marshall, D.A., and Wordsworth, S. (2018). Valuation of Health and Nonhealth Outcomes from Next-Generation Sequencing: Approaches, Challenges, and Solutions. Value Health *21*, 1043–1047. https://doi.org/10.1016/j.jval.2018.06.010.
36. Marshall, D.A., MacDonald, K.V., Heidenreich, S., Hartley, T., Bernier, F.P., Gillespie, M.K., McInnes, B., Innes, A.M., Armour, C.M., and Boycott, K.M. (2019). The value of diagnostic testing for parents of children with rare genetic diseases. Genet. Med. *21*, 2798–2806. https://doi.org/10.1038/s41436-019-0583-1.
37. Goranitis, I., Best, S., Stark, Z., Boughtwood, T., and Christodoulou, J. (2021). The value of genomic sequencing in complex pediatric neurological disorders: a discrete choice experiment. Genet. Med. *23*, 155–162. https://doi.org/10.1038/s41436-020-00949-2.

38. Meng, Y., Best, S., Amor, D.J., Braden, R., Morgan, A.T., and Goranitis, I. (2024). The value of genomic testing in severe childhood speech disorders. Eur. J. Hum. Genet. *32*, 440–447. https://doi.org/10.1038/s41431-024-01534-w.
39. Goranitis, I., Best, S., Christodoulou, J., Boughtwood, T., and Stark, Z. (2021). Preferences and values for rapid genomic testing in critically ill infants and children: a discrete choice experiment. Eur. J. Hum. Genet. *29*, 1645–1653. https://doi.org/10.1038/s41431-021-00874-1.
40. Dabbous, O., Chachoua, L., Aballéa, S., Sivignon, M., Persson, U., Petrou, S., Richardson, J., Simoens, S., and Toumi, M. (2023). Valuation of Treatments for Rare Diseases: A Systematic Literature Review of Societal Preference Studies. Adv. Ther. *40*, 393–424. https://doi.org/10.1007/s12325-022-02359-z.
41. Reckers-Droog, V., van Exel, J., and Brouwer, W. (2021). Willingness to Pay for Health-Related Quality of Life Gains in Relation to Disease Severity and the Age of Patients. Value Health *24*, 1182–1192. https://doi.org/10.1016/j.jval.2021.01.012.
42. Reckers-Droog, V., van Exel, J., and Brouwer, W. (2019). Equity Weights for Priority Setting in Healthcare: Severity, Age, or Both? Value Health *22*, 1441–1449. https://doi.org/10.1016/j.jval.2019.07.012.
43. Peasgood, T., Howell, M., Raghunandan, R., Salisbury, A., Sellars, M., Chen, G., Coast, J., Craig, J.C., Devlin, N.J., Howard, K., et al. (2024). Systematic Review of the Relative Social Value of Child and Adult Health. Pharmacoeconomics *42*, 177–198. https://doi.org/10.1007/s40273-023-01327-x.
44. Goldenberg, A.J., and Sharp, R.R. (2012). The Ethical Hazards and Programmatic Challenges of Genomic Newborn Screening. JAMA *307*, 461–462. https://doi.org/10.1001/jama.2012.68.
45. Nisselle, A., Bishop, M., Charles, T., Morrissy, S., King, E., Metcalfe, S., and Gaff, C. (2019). Lessons learnt from implementing change in newborn bloodspot screening processes over more than a decade: Midwives, genetics and education. Midwifery *79*, 102542. https://doi.org/10.1016/j.midw.2019.102542.
46. Tutty, E., Archibald, A.D., Downie, L., Gaff, C., Lunke, S., Vears, D.F., Stark, Z., and Best, S. (2024). Key informant perspectives on implementing genomic newborn screening: a qualitative study guided by the Action, Actor, Context, Target, Time framework. Eur. J. Hum. Genet. *32*, 1599–1605. https://doi.org/10.1038/s41431-024-01650-7.
47. Goranitis, I., Meng, Y., Martyn, M., Best, S., Bouffler, S., Bombard, Y., Gaff, C., and Stark, Z. (2024). Eliciting parental preferences and values for the return of additional findings from genomic sequencing. NPJ Genom. Med. *9*, 10–17. https://doi.org/10.1038/s41525-024-00399-8.
48. Bombard, Y., Ginsburg, G.S., Sturm, A.C., Zhou, A.Y., and Lemke, A.A. (2022). Digital health-enabled genomics: Opportunities and challenges. Am. J. Hum. Genet. *109*, 1190–1198. https://doi.org/10.1016/j.ajhg.2022.05.001.
49. Bombard, Y., Clausen, M., Mighton, C., Carlsson, L., Casalino, S., Glogowski, E., Schrader, K., Evans, M., Scheer, A., Baxter, N., et al. (2018). The Genomics ADvISER: development and usability testing of a decision aid for the selection of incidental sequencing results. Eur. J. Hum. Genet. *26*, 984–995. https://doi.org/10.1038/s41431-018-0144-0.
50. Lunke, S., Bouffler, S.E., Downie, L., Caruana, J., Amor, D.J., Archibald, A., Bombard, Y., Christodoulou, J., Clausen, M., De Fazio, P., et al. (2024). Prospective cohort study of genomic newborn screening: BabyScreen+ pilot study protocol. BMJ Open *14*, e081426. https://doi.org/10.1136/bmjopen-2023-081426.
51. Shickh, S., Rafferty, S.A., Clausen, M., Kodida, R., Mighton, C., Panchal, S., Lorentz, J., Ward, T., Watkins, N., Elser, C., et al. (2021). The role of digital tools in the delivery of genomic medicine: enhancing patient-centered care. Genet. Med. *23*, 1086–1094. https://doi.org/10.1038/s41436-021-01112-1.
52. Paquin, R.S., Peinado, S., Lewis, M.A., Biesecker, B.B., Rini, C., Roche, M., Butterfield, R.M., Powell, C.M., Berg, J.S., and Bailey, D.B. (2021). A behavior-theoretic evaluation of values clarification on parental beliefs and intentions toward genomic sequencing for newborns. Soc. Sci. Med. *271*, 112037. https://doi.org/10.1016/j.socscimed.2018.11.017.
53. Peinado, S., Paquin, R.S., Rini, C., Roche, M., Butterfield, R. M., Berg, J.S., Powell, C.M., Bailey, D.B., and Lewis, M.A. (2020). Values clarification and parental decision making about newborn genomic sequencing. Health Psychol. *39*, 335–344. https://doi.org/10.1037/hea0000829.
54. Newson, A. (2006). Should Parental Refusals of Newborn Screening Be Respected? Camb. Q. Healthc. Ethics *15*, 135–146. https://doi.org/10.1017/S0963180106060166.
55. Evans, J.R., and Mathur, A. (2005). The value of online surveys. Internet Res. *15*, 195–219. https://doi.org/10.1108/10662240510590360.
56. Evans, J.R., and Mathur, A. (2018). The value of online surveys: a look back and a look ahead. Internet Res. *28*, 854–887. https://doi.org/10.1108/IntR-03-2018-0089.
57. Quaife, M., Terris-Prestholt, F., Di Tanna, G.L., and Vickerman, P. (2018). How well do discrete choice experiments predict health choices? A systematic review and meta-analysis of external validity. Eur. J. Health Econ. *19*, 1053–1066. https://doi.org/10.1007/s10198-018-0954-6.
58. Kirk, E.P., Delatycki, M.B., Archibald, A.D., Tutty, E., Caruana, J., Halliday, J.L., Lewis, S., McClaren, B.J., Newson, A. J., Dive, L., et al. (2024). Nationwide, Couple-Based Genetic Carrier Screening. N. Engl. J. Med. *391*, 1877–1889. https://doi.org/10.1056/NEJMoa2314768.
59. Stark, Z., Glazer, D., Hofmann, O., Rendon, A., Marshall, C. R., Ginsburg, G.S., Lunt, C., Allen, N., Effingham, M., Hastings Ward, J., et al. (2025). A call to action to scale up research and clinical genomic data sharing. Nat. Rev. Genet. *26*, 141–147. https://doi.org/10.1038/s41576-024-00776-0.
60. Madden, E.B., Hindorff, L.A., Bonham, V.L., Akintobi, T.H., Burchard, E.G., Baker, K.E., Begay, R.L., Carpten, J.D., Cox, N.J., Di Francesco, V., et al. (2024). Advancing genomics to improve health equity. Nat. Genet. *56*, 752–757. https://doi.org/10.1038/s41588-024-01711-z.
61. Fatumo, S., Chikowore, T., Choudhury, A., Ayub, M., Martin, A.R., and Kuchenbaecker, K. (2022). A roadmap to increase diversity in genomic studies. Nat. Med. *28*, 243–250. https://doi.org/10.1038/s41591-021-01672-4.
62. Baynam, G., Julkowska, D., Bowdin, S., Hermes, A., McMaster, C.R., Prichep, E., Richer, É., van der Westhuizen, F.H., Repetto, G.M., Malherbe, H., et al. (2024). Advancing diagnosis and research for rare genetic diseases in Indigenous peoples.

Nat. Genet. *56*, 189–193. https://doi.org/10.1038/s41588-023-01642-1.

63. Skantharajah, N., Baichoo, S., Boughtwood, T.F., Casas-Silva, E., Chandrasekharan, S., Dave, S.M., Fakhro, K.A., Falcon de Vargas, A.B., Gayle, S.S., Gupta, V.K., et al. (2023). Equity, diversity, and inclusion at the Global Alliance for Genomics and Health. Cell Genom. *3*, 100386. https://doi.org/10.1016/j.xgen.2023.100386.

The American Journal of Human Genetics, Volume *112*

Supplemental information

Public preferences for the value and implementation of genomic newborn screening: Insights from two discrete choice experiments in Australia

Riccarda Peters, Stephanie Best, Fiona Lynch, Danya F. Vears, Lilian Downie, Alison D. Archibald, Sebastian Lunke, Zornitza Stark, and Ilias Goranitis

Supplemental Tables

Table S1. Summary of participant characteristics

	Value DCE		Implementation DCE		
	**Count (n)**	**% of Total**	**Count (n)**	**% of Total**	**Census Data 2021***
**Gender**					
Male	736	48.9%	492	49%	49.3%
Female	768	51.1%	513	51%	50.7%
Total	1504		1005		
**Age**	mean= 46.6	SD= 16.9	Mean= 48	SD= 18.1	
**Age bands**					
18-24 years	176	11.7%	113	11.2%	12%
25-34 years	279	18.6%	183	18.2%	19%
35-44 years	272	18.1%	166	16.5%	18%
45-54 years	256	17.0%	166	16.5%	17%
55-64 years	242	16.1%	153	15.2%	16%
65-74 years	196	13.0%	126	12.5%	13%
75+ years	83	5.5%	98	9.8%	6%
**State**					
NSW	467	31.1%	319	31.7 %	34.5%
VIC	392	26.1%	258	25.7 %	27.8%
QLD	285	18.9%	205	20.4 %	22.0%
SA	115	7.6%	74	7.4 %	7.6%
WA	161	10.7%	101	10.0 %	11.4%
ACT	35	2.3%	22	2.2 %	1.9%
TAS	34	2.3%	17	1.7 %	2.3%
NT	15	1.0%	9	0.9 %	0.99%
**Region**					
Metro	1104	73.4%	730	72.60%	66.9%
Regional	400	26.6%	275	27.40%	33.1%
**Income**					
Lower than $40k per year	218	14.5%	175	17.4%	11.4%
$40k - $60k per year	203	13.5%	137	13.6%	11.7%
$60,000 - $80,000 per year	188	12.5%	143	14.2%	13%
$80,000 - $100,000 per year	195	13.0%	133	13.2%	11%
$100,000 - $120,000 per year	182	12.1%	114	11.3 %	11.2%
$120,000 - $140,000 per year	113	7.5%	71	7.1%	7.1%
$140,000 - $160,000 per year	139	9.2%	92	9.2%	6.2%
Over $160,000 per year	266	17.7%	140	13.9%	22.5%
**Education**					
Year 11 or below	161	10.7%	110	10.9%	
Year 12 or equivalent	245	16.3%	170	16.9%	

Certificate	194	12.9%	160	15.9%	
Diploma/advanced diploma	184	12.2%	127	12.6%	
Bachelor's degree	442	29.4%	266	26.5%	
Graduate diploma/certificate	82	5.5%	51	5.1%	
Post-graduate degree	192	12.8%	119	11.8%	
Other (please specify):	4	0.3%	2	0.2%	
**Health Insurance**					
Yes	1037	68.9%	667	66.4%	
No	467	31.1%	338	33.6%	
**Current marital status**					
Never married	403	26.8%	278	27.7%	
De facto- living with a partner	220	14.6%	138	13.7%	
Married	701	46.6%	450	44.8%	
Widowed	43	2.9%	31	3.1%	
Divorced/separated	133	8.8%	105	10.4%	
Other	4	0.3%	3	0.3%	
**Do you have children**					
Yes	931	61.9%	633	63.0%	
No	573	38.1%	372	37.0%	
**Are you or your partner currently pregnant?**					
Yes	76	5.1%	50	5.0%	
No	1344	89.4%	859	85.5%	
Prefer not to say	21	1.4%	14	1.4%	
Not applicable	63	4.2%	82	8.2%	
**Do you plan to have children in the future**					
Yes	430	28.6%	281	28.0%	
No	831	55.3%	584	58.1%	
Not sure	236	15.7%	132	13.1%	
Prefer not to say	7	0.5%	8	0.8%	
**Do you or any of your close family members or friends have a genetic condition?**					**Across both surveys**
Yes	248	16.5%	161	16.0%	16%
No	1068	71.0%	668	66.5%	69%
Do not know	188	12.5%	176	17.5%	15%
**Have you or any of your close family members or friends ever had a genetic or genomic test?**					
Yes	309	20.5%	194	19.3%	20%
No	933	62.0%	645	64.2%	63%
Do not know	262	17.4%	166	16.5%	17%
**Have you read or heard about genomic testing before receiving this survey?**					
Yes	636	42.3%	414	41.2%	42%
No	868	57.7%	591	58.8%	58%

**How much did you know about newborn screening before receiving the invitation to participate in this survey?**					
I had never heard of newborn screening	284	18.9%	198	19.7%	19%
I had heard of newborn screening, but I did not know anything about it	470	31.3%	320	31.8%	31%
I knew a little about newborn screening	656	43.6%	421	41.9%	43%
I knew a lot about newborn screening	94	6.3%	66	6.6%	6%
**How familiar do you feel with how genetic conditions affect people's lives and the lives of those around them?**					
0-10	mean= 5.32	SD= 2.53	mean= 5.29	SD= 2.66	
**Overall, how comfortable do you feel you are in taking risks regarding your health?**					
0-10	mean= 5.23	SD= 2.5	mean= 5.24	SD= 2.59	
**How often do you need to have someone help you when you read instructions, pamphlets, or other written material from your doctor or pharmacy?**					
(Never) 1	894	59.4%	579	57.6%	
2	223	14.8%	135	13.4%	
3	189	12.6%	146	14.5%	
4	159	10.6%	110	10.9%	
(Always) 5	39	2.6%	35	3.5%	

*Note: Australian data were sourced from the Australian Bureau of Statistics online, see www.abs.gov.au.

Table S2. Indications of preferences for age-of onset of conditions and consent for Genomic Newborn Screening (GNBS)

**Which of the following types of results from genomic newborn screening would you like to receive for your baby? (n= 1504)**	Count (n)	% of Total
Infancy: less than 1 year old	1082	71.9%
Early childhood: between 1-5 years	731	48.6%
Late childhood: between 6-12 years	485	28.5%
Adolescence: between 13- 18 years	482	28.5%
Adulthood	349	23.2%
None at all	151	10%
**Preferred method of consent for GNBS (n= 1004)**		
Opt-in	655	65.2%
Opt-out	109	10.8%
Implied consent	241	24.0%

Note: Participants in the value DCE (n= 1504) were asked for which age group they would prefer to receive results of GNBS. Multiple Selections were allowed; Participants in the implementation DCE (n= 1004) were asked to indicate their preferred method of consent for GNBS, only one selection was allowed.

Table S3. Marginal utilities based on the latent class choice model for the value DCE

	Attributes	Class 1	Class 2	Class 3	Class 4
**Severity of condition**	Profound conditions only	base	base	base	base
	Profound & moderate conditions	0.38826	0.70428	1.56031[a]	0.19997
	Profound, moderate & mild conditions	-0.39764	0.51668	0.56994[a]	0.68417[a]
**Certainty that the condition develops**	High, moderate & average certainty (greater than 50%)	base	base	base	base
	High & moderate certainty (greater than 75%)	-2.75468	0.10157	-0.11794	0.14015
	High certainty only (greater than 90%)	-1.37765	0.36861	-0.17374	-0.03385
**Treatment availability**	Treatments that cure conditions	base	base	base	base
	Treatments that cure conditions or manage their symptoms	0.55164	0.29652	-0.27285	0.37214[b]
	Treatments that cure conditions or manage their symptoms & conditions without current treatment	1.38633	-0.19271	0.54041[a]	0.27376
**Additional number of newborns diagnosed compared to standard newborn screening (in every 1000 newborns screened)**		-0.04374	0.01436	0.00448	0.02545[a]
**Accuracy of screening results**	95%	base	base	base	base
	98%	-1.46974	0.52225[a]	0.50858[a]	0.28094[b]
	100%	1.22888	0.76369[a]	1.15912[a]	0.65573[a]
**Cost of testing to you (AU$)**		-0.00048	-0.00139[a]	0.00010	-0.00143[a]
**Additional diagnoses x Profound & moderate conditions**		-0.06762	-0.0103	0.00073	-0.01438
**Additional diagnoses x Profound, moderate & mild conditions**		-0.0757	-0.02427[b]	0.00956	-0.02432[a]
**Additional diagnoses x High & moderate certainty (greater than 75%)**		0.14023	-0.00233	-0.00089	-0.00411
**Additional diagnoses x High certainty only (greater than 90%)**		0.18289	-0.02176[b]	0.00449	0.01692[b]
**Additional diagnoses x Treatments that cure conditions or manage their symptoms**		-0.06662	0.00762	0.01575[a]	-0.00747
**Additional diagnoses x Treatments that cure conditions or manage their symptoms & conditions without current treatment**		-0.10166	0.00998	-0.00374	-0.01104
**Genomic newborn screening constant**		-4.32240[a]	4.35961[a]	2.18950[a]	0.36227
**Class probabilities**		13%	28%	42%	17%
**Log Likelihood Function**	-8093.4962				
**McFadden Pseudo R-Squared**	0.3877146				
**Akaike information criterion**	16329				

[a] Statistically significant at 1% level; [b] Statistically significant at 5% level.

Table S4. Fractional logistic regression to explore preference heterogeneity for the probability of choosing GNBS in the Value DCE

	**Class 1**	**Class 2**	**Class 3**	**Class 4**
**Age**<br>(being 45+ years)	0.4887[b]	-0.2013	0.0686	-0.2173
**Gender**<br>(being female)	0.2355	-0.2202[b]	-0.0124	0.1607
**Education**<br>(having University degree or higher)	-0.3980[b]	0.0322	0.1489	0.0032
**Income**<br>(having and income over AU$100k per year)	-0.5204[b]	0.0189	0.1595	0.0518
**Region**<br>(living in a metropolitan area)	-0.0473	-0.0890	0.1723	-0.1268
**Marital status**<br>(being married or in a de facto relationship)	-0.2511	0.0592	0.2300	-0.2453
**Children**<br>(having children)	0.4284[b]	0.0413	-0.3851[a]	0.2093
**Having experience with a genetic condition**	-0.7460[a]	-0.0933	0.3700[b]	-0.1046
**Having experience with a genetic test**	-0.1071	0.1103	-0.1221	0.0689
**Having heard about genomic testing before**	0.1373	0.0192	0.0285	-0.1718
**Having knowledge about genetic conditions**	-0.0926[a]	0.0279	0.0648[a]	-0.0598
**Having knowledge about newborn screening**	-0.1099	0.0055	0.0222	0.0396
**Bering comfortable in taking health risks**	-0.0549	0.0020	0.0405	-0.0247
**Having higher health literacy**	-0.0345	-0.0458	0.1194[b]	-0.0914

[a] Statistically significant at 1% level; [b] Statistically significant at 5% level.

Table S5. Marginal utilities including sociodemographic x attribute interactions for the Implementation DCE

**Attribute**	**Level**	**Mean**	**Standard Error**
When is GNBS first discussed?	First doctor appointment		
	Second trimester	0.12795	0.08015
	Third trimester	0.11097	0.06982
	Shortly after birth	0.11515	0.06921
Who provides initial information	A midwife or nurse		
	Your GP	0.15724[b]	0.06928
	An obstetrician	0.14103[b]	0.067
	A genetic health professional	0.07821	0.05685
What support material is available?	No support material		
	Leaflet	0.11370	0.06615
	Interactive online portal	0.08481	0.07007
	Appointment with health professional	0.1097	0.07116
What conditions are included?	Selected by parents		
	Selected by health professionals	0.12191	0.0754
Who returns “high chance” results?	Your GP		
	A relevant medical specialist	0.10931	0.07453
	A genetic health professional	0.19811[b]	0.0814
How are “high chance” results returned?	Online portal		
	Telehealth or phone	0.24372[a]	0.07761
	In person	0.28376[a]	0.06691
How are “low chance” results returned?	No return of low chance results		
	Online portal	0.08178	0.0635
	Telehealth or phone	0.08267	0.07013
	In person	0.29958[a]	0.08583
What happens if new relevant information becomes available?	No updates will be provided		
	Updates on request	0.10576	0.07291
	Provided automatically in secure online portal	0.0917	0.07521
GNBS constant (random parameter)	GNBS	4.06009[a]	0.2802
**Sociodemographic interacted with attributes:**			
Income (high) x GNBS should be discussed in second trimester		-0.15793	0.08287
Age (over 45) x Initial information provided by an obstetrician		-0.0825	0.08835

Age (over 45) x low chance results returned in person		-0.13633	0.07795
Education (university or higher) x low chance results returned in person		-0.17894[b]	0.07878
Log Likelihood Function	-6723.78552		
McFadden Pseudo R-Squared	0.2387745		
Akaike information criterion	13499.6		

[a] Statistically significant at 1% level; [b] Statistically significant at 5% level.

Supplemental Methods

Checklist for reporting discrete choice experiments in health: the DIRECT Checklist

Section<br><br>Item		Page and paragraph
Purpose and rationale		
1	Describe the real-world context and decision-maker that the hypothetical choice context seeks to replicate or inform	p.4|para.4
2	Provide a rationale for using a DCE to answer the research question	p.4|para.3
Attributes and levels[a]		
3	Describe how attributes and levels were derived (e.g. literature review, interviews, focus groups, expert input)	p.5|para.2
4	Provide the final list of attributes and levels	Table 1 & 2
Experimental design		
5	Report the number of alternatives per choice set and whether they were labelled or unlabelled	Appendix A,p.23|para.2
6	Describe response options (e.g. forced choice, opt-out, status quo)	Appendix A, p.23|para.2
7	Describe the type of experimental design (e.g. orthogonal, D-efficient, Bayesian efficient, partial profile)	Appendix A, p.23|para.2
8	Describe which effects are identified in the design (e.g. main effects, higher order interactions, functional form)	Appendix A,p.23|para.2
9	Describe the number of choice sets, blocks and choice sets per block	Appendix A, p.23|para.3
10	Indicate how the experimental design was obtained (software, catalogue, other)	Appendix A, p.23|para.3
Survey design		
11	Provide a sample choice set and the instructions and background information given to respondents (e.g. providing the survey as an appendix)	Surveys in supplementary material
12	Report any randomisation (e.g. choice set order, attribute order, alternative order, framing effects)	Appendix A, p.24|para.2
13	Describe what was checked in piloting (e.g. understanding, respondent burden, timing, wording)	Appendix A, p.24|para.3
14	Report whether information from the pilot was used to update the experimental design (e.g. priors, functional form of attributes) or survey design	Appendix A, p.24|para.3
Sample and data collection		
15	Report respondent inclusion/exclusion criteria	Appendix A,p.24|para.4
16	Describe how data were collected (e.g. mail, personal interview, web survey)	Appendix A, p.24|para.4
17	Report the response rate or cooperation rate, if possible	N/A
18	Report the final sample size and how the sample size was determined	p.7|para.1
19	Describe respondent characteristics and representativeness of target population, if known	Table S1
Econometric analysis		
20	Indicate coding of data (e.g. effects, dummy, continuous) including definitions	Appendix A, p.25|para.3
21	Report whether any respondents were removed and why (e.g. suspected fraudulent responses, rationality tests)	N/A

Section<br><br>Item		Page and paragraph
22	Provide the rationale for model choice (e.g. conditional logit, mixed logit, latent class) and assumptions (e.g. error variance)	Appendix A, p.25|para.2
23	Report model specification	Appendix A,p.25|para.3
Reporting of results		
24	Report the model performance, goodness of fit (if comparing models)	Within tables
25	Describe methods used for analysis of model results (e.g. calculation of marginal rate of substitution, attribute relative importance, welfare gain)	Appendix A, p.25|para.4
26	Report measures of precision for the output(s) of interest (e.g. confidence intervals) and how these were derived	Appendix A, p.25|para.4

DIRECT reporting checklist for the reporting of the value and implementation.

Study design and participants

The DCE surveys were designed following best practice recommendations for development,[1] analysis[2] and reporting.[3] As recommended,[4] focus groups were conducted to identify and develop the DCE attributes, and detailed information about the focus groups methods and findings is available elsewhere.[5,6] Attribute levels were identified in consultation with the genetic experts of the research team to ensure clinical face validity. The final attributes and corresponding levels for both DCEs are shown in **Tables 1 and 2** Labelled D-efficient partial profile designs were developed using Ngene (ChoiceMetrics [2018] Ngene 1.2 User Manual & Reference Guide, Australia). Participants were presented with two gNBS alternatives and an opt-out option representing the standard NBS. For the value DCE, explicit partial profiles with two overlapping attributes were chosen to reduce task complexity and avoid dominance issues.[7] In each choice task, two of the three attributes related to the conditions included in the screening program were kept the same (overlapping) in a choice task, while varying the levels of the remaining six attributes. Which attribute levels were overlapping varied across choice tasks. Restrictions were applied to ensure the attribute combinations were realistic. For example, if more conditions were included due to less stringent inclusion criteria, this would result in diagnosing more children and a higher test cost. For the implementation DCE, an implicit partial profile design was used, where only four attributes were shown to participants (i.e., the other four overlapping attributes were not displayed). Different overlapping attributes or levels were used across choice tasks. An example of a choice task for the value and implementation DCEs is shown in **Figures 1 and 2**, respectively.

The experimental design for the value DCE included 32 choice tasks split across four blocks; and 64 choice tasks split across 6 blocks for the implementation DCE. Thus, for both surveys each participant required to complete 8 choice tasks. For both studies choice tasks were drawn from a candidate set of relevant attribute combinations using the modified Federov algorithm.

Restrictions were applied to the algorithm to ensure fair representation of all attribute levels without imposing attribute level balance.

Several randomizations were used. Participants were first randomly assigned to different choice blocks. Then, the order of choice tasks within blocks and the order of the two gNBS situations were randomized to avoid any left-to-right bias. To check for internal consistency in the value DCE, a dominant choice set was included as a ninth-choice task for each participant, which was excluded from the modelling.

The value DCE survey was reviewed by a plain language advisor and piloted using a think aloud interview amongst a convenience sample of university staff to assess completion time, language and understanding (n=5). Prior to the full launch of the DCE surveys, the surveys underwent several rounds of piloting (n= 873) to evaluate if the coefficients were logically ordered, to assess completion time, and to gather feedback on the difficulty of the task. The pilot results were used to refine the DCE experimental design and final survey wording. To ensure participants were adequately prepared with information to complete the surveys, the final surveys included an educational component, which comprised a video developed by the Babyscreen+ study team to explain genomic newborn screening. Participants were further presented with an explanation of genomic newborn screening characteristics and were guided through an explanation of the DCE with an example. The surveys commenced with screening questions to check for eligibility, followed by demographic questions, including questions about parental status, experience with genetic conditions, risk attitudes and knowledge about health. The final surveys and the movie used as educational material are available in the **Supplemental material.**

In accordance with recommendations from health economics on the measurement and valuation of health benefits for economic evaluation,[8] we sought the values of the general public, who are both taxpayers and potential users of healthcare. Two independent Australia-wide sample of participants over the age of 18 years were recruited from nationwide panels through the research market company Pureprofile. Participants completed the survey online. Age, gender,

income and geographical location (states) quotas were used to ensure that the sample was representative of the Australian public. This was further validated against other national sources.[9]

Ethics Statement

Informed consent was obtained from all participants before entering the survey. Ethics approval was granted from the Royal Children's Hospital Melbourne Human Research Ethics Committee (Ethics ID: HREC/91392/RCHM-2022).

Choice Analysis

Choice data were analyzed using a panel error component mixed logit model, which uses random parameters to account for unobserved heterogeneity of preferences among participants.[2]

In the value DCE, continuous coding was applied for the attributes "Additional number of newborns diagnosed (in every 1000 newborns screened)" and "Cost of testing to you", with the remaining attributes being dummy coded. Normal distributions were used for all parameters apart from cost, for which a triangular distribution was used.[10] Interactions between the attribute "Additional number of newborns diagnosed compared to standard newborn screening" and the attributes "Severity of conditions", "Certainty that the condition develops", and "Treatment availability for conditions screened" were included as main effects in the model, due to the restrictions imposed in the design.[11] Attributes in the implementation DCE were dummy coded and normal distributions were used for all parameters. Random parameters were estimated using 1000 standard Halton sequences.

We estimate the relative importance of each attribute based on the proportional change in overall utility associated with each attribute.[12] Finally we estimate the overall value that the public attaches to a publicly-funded gNBS with a restrictive and non-restrictive gNBS implementation relative to standard NBS using the compensating variation method[13], which was applied using the Wald method. Estimates account for attribute interactions and CIs were estimated using the

Delta method. The values are reported in both Australian and US dollars (using 21 November 2024 Reserve Bank of Australia exchange rate of 0.65).

To further explore participants' heterogeneity in responses in the value DCE, we utilized a latent class (LC) choice model,[14] which divides the sample into a finite number of groups (classes) with homogenous preferences. Using the Akaike information criterion (AIC), we estimated a model with 4 classes. A fractional logistic regression was then used to explore whether sociodemographic and attitudinal characteristics were significantly associated with class membership probabilities.[15] The results of the latent class model were used to provide an estimate of the uptake of gNBS based on the proportion of participants demonstrating no preference for gNBS and its attributes. To explore drivers of response heterogeneity in the implementation DCE, we extended the base case model to explore potential drivers of heterogeneity. Sociodemographic characteristics entered the model as interactions with GNBS attributes that showed significant heterogeneity as evidenced by significant standard deviations. We undertook an iterative process examining the variable's statistical significance as a prediction of choice under each specification. This approach to preference heterogeneity analysis was preferred given the implicit partial profile design adopted in this DCE.

Panel error component mixed logit models, LC choice model and the Wald method were conducted in NLOGIT 6 (Econometric Software, Inc., Waverton, NSW, Australia) all other analyses were performed in Stata 16 (StataCorp, College Station, TX, USA).

Supplemental References

1. Reed Johnson, F., Lancsar, E., Marshall, D., Kilambi, V., Mühlbacher, A., Regier, D.A., Bresnahan, B.W., Kanninen, B., and Bridges, J.F.P. (2013). Constructing Experimental Designs for Discrete-Choice Experiments: Report of the ISPOR Conjoint Analysis Experimental Design Good Research Practices Task Force. Value in Health *16*, 3–13. https://doi.org/10.1016/j.jval.2012.08.2223.

2. Hensher, D.A., Rose, J.M., and Greene, W.H. (2015). Applied Choice Analysis 2nd ed. (Cambridge University Press) https://doi.org/10.1017/CBO9781316136232.

3. Ride, J., Goranitis, I., Meng, Y., LaBond, C., and Lancsar, E. (2024). A Reporting Checklist for Discrete Choice Experiments in Health: The DIRECT Checklist. PharmacoEconomics *42*, 1161–1175. https://doi.org/10.1007/s40273-024-01431-6.

4. Coast, J., and Horrocks, S. (2007). Developing attributes and levels for discrete choice experiments using qualitative methods. J Health Serv Res Policy *12*, 25–30. https://doi.org/10.1258/135581907779497602.

5. Lynch, F., Best, S., Gaff, C., Downie, L., Archibald, A.D., Gyngell, C., Goranitis, I., Peters, R., Savulescu, J., Lunke, S., et al. (2024). Australian Public Perspectives on Genomic Newborn Screening: Risks, Benefits, and Preferences for Implementation. International Journal of Neonatal Screening *10*, 6. https://doi.org/10.3390/ijns10010006.

6. Lynch, F., Best, S., Gaff, C., Downie, L., Archibald, A.D., Gyngell, C., Goranitis, I., Peters, R., Savulescu, J., Lunke, S., et al. (2024). Australian public perspectives on genomic newborn screening: which conditions should be included? Human Genomics *18*, 45. https://doi.org/10.1186/s40246-024-00611-x.

7. Kessels, R., Jones, B., and Goos, P. (2011). Bayesian optimal designs for discrete choice experiments with partial profiles. Journal of Choice Modelling *4*, 52–74. https://doi.org/10.1016/S1755-5345(13)70042-3.

8. Brazier, J., Ratcliffe, J., Saloman, J., and Tsuchiya, A. (2016). Measuring and Valuing Health Benefits for Economic Evaluation (Oxford University Press) https://doi.org/10.1093/med/9780198725923.001.0001.

9. Australian Bureau of Statistics (ABS). 2021 Census All persons QuickStats. (2021). https://www.abs.gov.au/census/find-census-data/quickstats/2021/AUS.

10. Daly, A., Hess, S., and Train, K. (2012). Assuring finite moments for willingness to pay in random coefficient models. Transportation *39*, 19–31. https://doi.org/10.1007/s11116-011-9331-3.

11. Nicolet, A., Groothuis-Oudshoorn, C.G.M., and Krabbe, P.F.M. (2018). Does Inclusion of Interactions Result in Higher Precision of Estimated Health State Values? Value in Health *21*, 1437–1444. https://doi.org/10.1016/j.jval.2018.06.001.

12. Gonzalez, J.M. (2019). A Guide to Measuring and Interpreting Attribute Importance. Patient *12*, 287–295. https://doi.org/10.1007/s40271-019-00360-3.

13. Small, K.A., and Rosen, H.S. (1981). Applied Welfare Economics with Discrete Choice Models. Econometrica *49*, 105–130. https://doi.org/10.2307/1911129.

14. Greene, W.H., and Hensher, D.A. (2003). A latent class model for discrete choice analysis: contrasts with mixed logit. Transportation Research Part B: Methodological *37*, 681–698. https://doi.org/10.1016/S0191-2615(02)00046-2.

15. Papke, L.E., and Wooldridge, J.M. (1996). Econometric Methods for Fractional Response Variables With an Application to 401 (K) Plan Participation Rates. Journal of Applied Econometrics *11*, 619–632.

Project: Australian public's perspectives on genomic newborn screening

Your say on Genomic Newborn Screening

Can you take part in our survey?

We want to hear your thoughts on genomic newborn screening. The newborn screening programs in Australia do not use genomic testing at present. But there is great potential for it to be used in the future. We want you to have a say on what this may look like. We have developed a survey that takes about 20 minutes to complete. You'll start by watching a quick video on genomic newborn screening. Afterward, we'll present you with a series of questions where you will have the opportunity to choose between different genomic newborn screening programs. Additionally, we'll ask for some basic information about you, like your age and gender.

What is newborn screening?

Every new baby has a test to screen for genetic conditions at birth. This is performed on a few drops of blood collected soon after birth by pricking the baby's heel, called the 'heel prick test'. Newborn screening looks for around 25 rare but serious health conditions in babies. These conditions can be life-threatening or affect development.

What is genomic newborn screening?

New technologies can be used in newborn screening to look for many more health conditions. One of these is genomic testing. This is a powerful tool we use to look for DNA changes that can impact health. To see these changes, we map out a person's whole genetic code. The DNA changes we find can reveal useful information about the chance of developing one or many different health conditions. It is possible to screen for many more treatable childhood-onset conditions using genomic testing. This is called genomic newborn screening

Your participation is voluntary. Your responses will be unidentifiable, confidential, and not shared with anyone outside the research team. This research has ethical approval from The Royal Children's Hospital Melbourne. You can withdraw from this research at any time and without any consequences.

Please click here to review the **Participant Information Statement**, where we describe the project and the nature of your participation.

If you have any questions, please contact Dr Riccarda Peters or A/Professor Ilias Goranitis.

Thank you for your time and help!

The consent process

By clicking the arrow to proceed, I am indicating my consent to participate in this study as outlined in the Plain Language Statement.

Screening questions

Before you proceed to the survey, we need to confirm you meet all the criteria below to take part. Please click the appropriate button below:

1. I am aged 18 or older

☐ Yes

☐ No

2. I am an Australian citizen or resident

☐ Yes

☐ No

3. I speak English

☐ Yes

☐ No

(*No to any 1-3 do not qualify*)

Demographics

About you

We will ask a few questions about you so we can better understand your answers.

1. How old are you? __________________ years

2. What is your gender:

☐ Female
☐ Male
☐ Prefer to self-describe __________________

3. What state or territory do you live in? Please check only one

☐ New South Wales
☐ Victoria
☐ Queensland
☐ South Australia
☐ Western Australia
☐ Tasmania
☐ Northern Territory
☐ Australian Capital Territory

4. What is your highest level of education?

☐ Year 11 or below
☐ Year 12 or equivalent
☐ Certificate
☐ Diploma/advanced diploma
☐ Bachelor's degree
☐ Graduate diploma/certificate
☐ Post-graduate degree
☐ Other (please specify): __________________

5. What is your household's gross annual income – that is, your earnings before tax?

☐ Lower than $40,000 per year
☐ $40,000 - $60,000 per year
☐ $60,000 - $80,000 per year
☐ $80,000 - $100,000 per year
☐ $100,000 - $120,000 per year
☐ $120,000 - $140,000 per year
☐ $140,000 - $160,000 per year
☐ Over $160,000 per year

6. Do you have private health insurance?

☐ Yes
☐ No

7. What is your current marital status?

☐ Never married
☐ De facto – living with a partner
☐ Married
☐ Widowed
☐ Divorced / separated
☐ Other (please specify): ___________________

8. Do you have children?

☐ Yes (if yes, go to 9)
☐ No (if no, go to 11)

9. How many children do you have? ________
10. How old is your youngest child? Age in years ________

11. Are you or your partner currently pregnant?

☐ Yes

☐ No
☐ Prefer not to say
☐ Not applicable

12. Do you plan to have children in the near future? Please check only one.

☐ Yes
☐ No
☐ Not sure
☐ Prefer not to say

Experience with genetic conditions and newborn screening

13. A genetic condition is a health issue caused by changes in our DNA that can affect our bodies or how we think and feel. Do you or any of your close family members or friends have a genetic condition? (e.g., Cystic Fibriosis)

☐ Yes
☐ No
☐ Do not know

14. A genetic or genomic test is a test that looks at our DNA to find out information about our genes and how they might affect our health. Have you or any of your close family members or friends ever had a genetic or genomic test?

☐ Yes
☐ No
☐ Do not know

15. Have you read or heard about genomic testing before receiving this survey?

☐ Yes
☐ No

16. How familiar do you feel with how genetic conditions affect people's lives and the lives of those around them?

Give a number between 0- 10. Here, 0 means ‘I am not familiar at all’. And 10 means ‘I know exactly what it means to have a genetic condition or to live with someone who has a genetic condition’:

____

17. How much did you know about newborn screening before receiving the invitation to participate in this survey? Please check only one.

☐ I had never heard of newborn screening
☐ I had heard of newborn screening but I did not know anything about it
☐ I knew a little about newborn screening
☐ I knew a lot about newborn screening

Attitudes towards risk

18. In general, people often face risks when making health, financial, career and other life decisions. Overall, how comfortable do you feel you are in taking risks regarding your health?

Give a number between 0- 10. Here, 0 means ’I am not comfortable at all’. And 10 means ‘I am extremely comfortable’:

____

Knowledge about health

19. How often do you need to have someone help you when you read instructions, pamphlets, or other written material from your doctor or pharmacy?
(5-point Likert-type scale, 1 = never to 5 = always)

Background

Please watch the short video below to learn more about newborn screening and genomic newborn screening.

https://player.vimeo.com/video/834581569

Genomic newborn screening characteristics

People may consider several factors when deciding to participate in genomic newborn screening.

One such factor may be the type of conditions included in the screening. The conditions may vary in how much they impact life expectancy and quality of life, the extent to which treatment is currently available to cure the condition or manage its symptoms, and the likelihood that the detected condition will in fact develop.

The type and number of conditions included in the screening determines the number of diagnoses made and the number of newborns that can benefit from appropriate therapies now or in the future. Other factors may include the cost of screening (if not covered by Medicare) and the accuracy of the diagnosis.

Some people may worry about the implications of screening on life insurance premiums, although life insurance companies have agreed to a Moratorium that limits the use of genetic test results when assessing applications for life insurance. More information about the Financial Services Council Moratorium on genetic tests in life insurance can be found here.

This section will provide some more information about these key characteristics of genomic newborn screening. Please read carefully as the next section will ask you to make choices based on how important these characteristics are to you.

Severity of condition (without treatment)

This characteristic tells you **how severe** the conditions included in the genomic newborn screening program are in the absence of treatment.

Mild

Conditions at this level typically have minimal impact on both quality of life and life expectancy. Individuals with mild genetic conditions often lead relatively normal lives with few significant disruptions.

Example: Baby James has a condition that means his kidneys aren't as efficient as they should be. He needs to visit the doctor twice a year to check his urine and blood. James has normal growth and development.

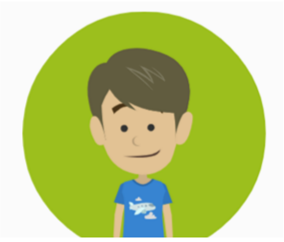

Moderate

Conditions may lead to more moderate effects on quality of life and could have a variable impact on life expectancy. Individuals with moderate genetic conditions may face challenges, but with appropriate management, they can maintain a reasonably good quality of life.

Example: Baby Aamira is diagnosed with a vision problem following newborn screening. Despite having glasses fitted and special drops for her eyes she still has very low vision and uses a guide dog in later childhood. The rest of her health and development are normal.

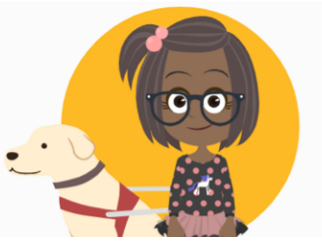

Profound

Conditions at this level significantly impact both quality of life and life expectancy. Individuals with profound genetic conditions often face substantial challenges, requiring comprehensive medical care and support in daily life. These conditions can affect life expectancy, potentially leading to death in childhood or early adulthood.

Example: Jonah has a condition where his body can't process food into energy adequately. He needs daily medication and a special, very restricted diet. He has to come into hospital if he gets even a common cold. He doesn't learn to walk or talk and has a severe intellectual disability.

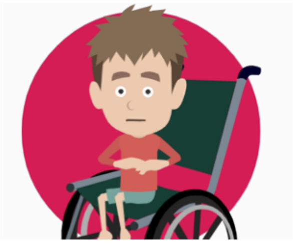

Certainty that the condition develops (without treatment)

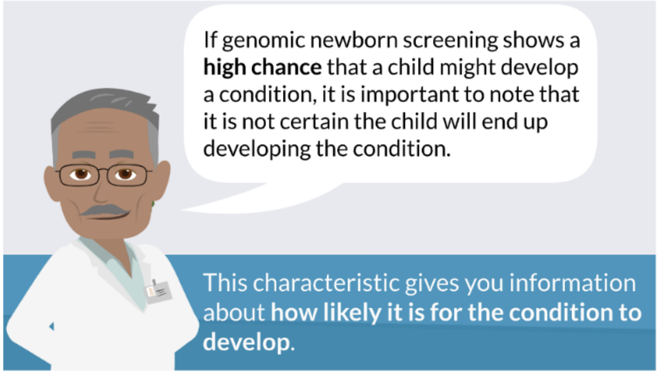

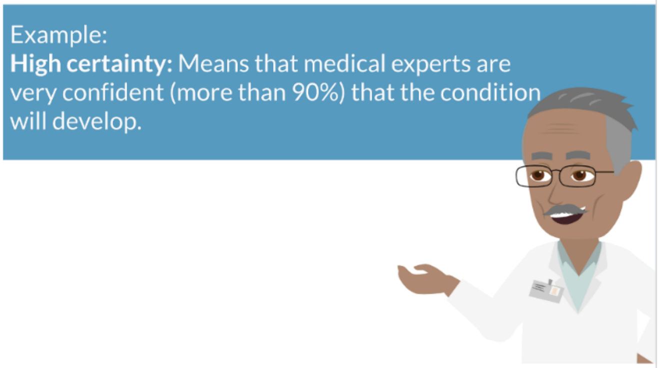

Treatment availability for conditions screened

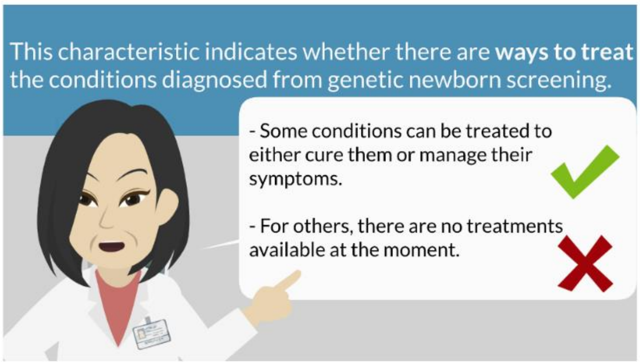

- Getting diagnosed early can provide valuable information about what to expect in the future.
- This can help with preparation and planning and save time searching for a diagnosis later even for conditions where there is no treatment available at the moment.
- It can also open opportunities to participate in research and clinical trials that contribute to medical knowledge and enhance patient care.
- Scientific knowledge is constantly developing and new treatments may become available in the future.

Additional number of newborns diagnosed
(in every 1000 newborns screened)

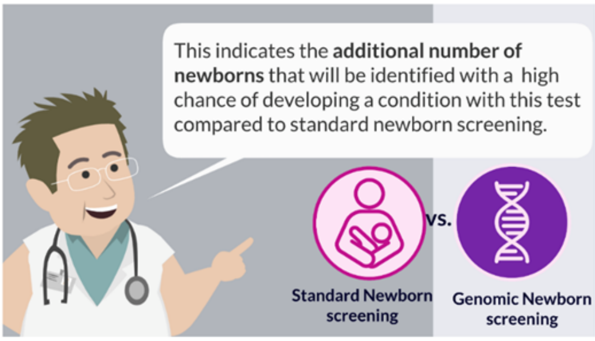

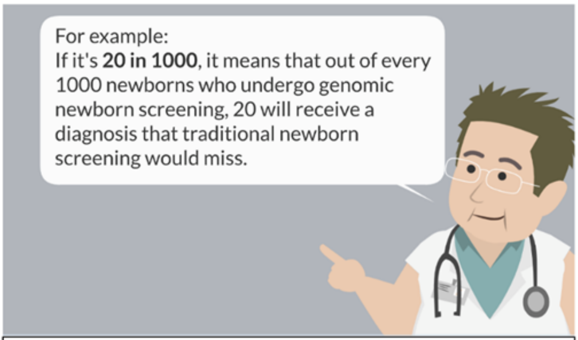

An early diagnosis offers great benefits for your baby, like the opportunity for early treatment to improve their health and potentially prevent severe problems or even death.

Accuracy of screening results

How likely it is that the result from newborn screening is **correct**.

Example:
95% accurate means that out of every 100 newborns labelled as 'high chance,' 95 of them will indeed have the condition, while 5 will not.

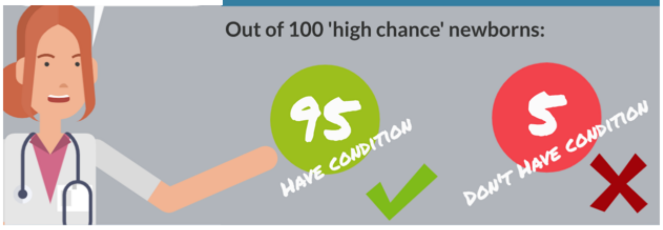

- If the screen identifies a baby has having a high chance of developing a health condition, parents will be referred to the right specialists to confirm the diagnosis and discuss treatment options.
- When the screening test gives a **wrong result**, it might lead to extra tests that parents might find:
  - Stressful
  - Anxiety-inducing
  - Confusing

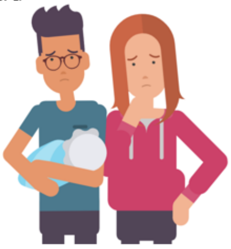

Cost of Genomic Newborn Screening to you

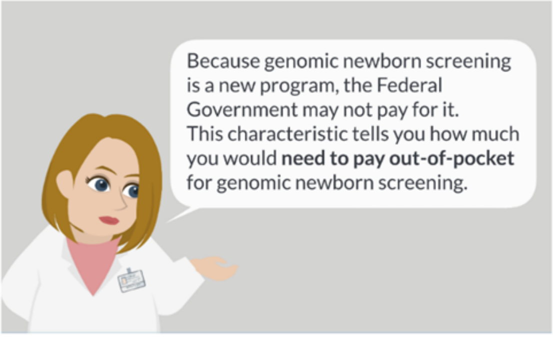

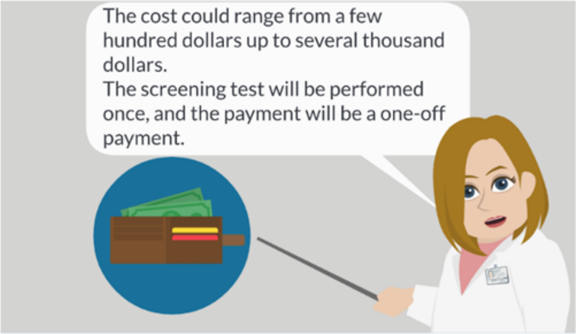

1. Age of onset

When a medical condition first starts affecting someone, it is referred to as the 'onset' of the condition. The onset is important, as it helps us understand when people are most likely to experience the first symptoms or signs of a particular condition.

- **Childhood-onset conditions** appear during childhood or adolescence.
- **Adult-onset conditions** tend to develop later in life, after a person becomes an adult.

For the following question imagine you had a newborn baby.

Which of the following types of results from genomic newborn screening would you like to receive for your baby? Tick all that apply.

Age of onset in:

a) Infancy: less than 1 year old
b) Early childhood: between 1-5 years
c) Late childhood: between 6-12 years
d) Adolescence: between 13- 18 years
e) Adulthood
f) None at all.

DCE explanation and example

In this section, we'll present two genomic screening programs side by side. These programs consist of the seven features we discussed earlier, and you can compare them.

Your task:

- Imagine you have a newborn and are offered the opportunity to take part in a genomic newborn screening program.
- Choose the newborn screening scenario that you would like the most for your child.
- If you would not choose either of the genomic newborn screening programs, select the option 'no genomic screening'. Your child would still have the standard 'heel prick test' that checks for 25 rare but serious childhood conditions. Since this is a public health initiative, you would not need to pay anything for standard newborn screening.

Example 1:

Now, let's go over an example together before we start the task.

The situation below has two genomic newborn screening programs ('Genomic newborn screening 1' and 'Genomic newborn screening 2') that are a bit different in some of the six characteristics we talked about before. Some of these characteristics are greyed out, this means they are the same across both options.

	Genomic Newborn Screening 1	Genomic Newborn Screening 2	Standard Newborn Screening only
**Severity of the condition**	Profound moderate & mild	Profound & moderate	
**Certainty that the condition develops**	High moderate & average certainty (greater than 50%)	High moderate & average certainty (greater than 50%)	
**Availability of treatment**	Treatments that cure conditions or manage their symptoms	Treatments that cure conditions or manage their symptoms	
**Additional number of newborns diagnosed compared to standard newborn screening (in every 1000 newborns screened)**	50 in 1000	20 in 1000	
**Accuracy of screening results**	100%<br>(none of the newborns screened will receive a wrong initial diagnosis)	95%<br>(5 out of 100 newborns screened will receive a wrong initial diagnosis)	
**Cost of genomic newborn screening to you**	$2,500	$1000	$0

I would prefer:

○ Genomic Newborn Screening 1

○ Genomic Newborn Screening 2

○ Standard Newborn Screening only

Example 1:

	Genomic Newborn Screening 1	Genomic Newborn Screening 2	Standard Newborn Screening only
**Severity of the condition**	Profound moderate & mild	Profound & moderate	
**Certainty that the condition develops**	High moderate & average certainty (greater than 50%)	High moderate & average certainty (greater than 50%)	
[illegible]	Treatments that cure conditions or manage their symptoms	Treatments that cure conditions or manage their symptoms	
**[illegible]orns [illegible]ndard [illegible]reened)**	50 in 1000	20 in 1000	
**Accuracy of screening results**	100% (none of the newborns screened will receive a wrong initial diagnosis)	95% (5 out of 100 newborns screened will receive a wrong initial diagnosis)	
**Cost of genomic newborn screening to you**	$2,500	$1000	$0

I would prefer: ○ Genomic Newborn Screening 1 ○ Genomic Newborn Screening 2 ○ Standard Newborn Screening only

Genomic Newborn Screening 1 includes also **mild conditions**, which means this option screens for **more** health conditions than Genomic Newborn Screening 2.

Rows that are grayed out mean that they are the same in both Genomic Newborn Screening 1 and Genomic Newborn Screening 2.

Example 1:

	Genomic Newborn Screening 1	Genomic Newborn Screening 2	Standard Newborn [illegible]
Severity of the condition	Profound moderate & mild	Profound & moderate	[illegible]
**Certainty that the condition develops**	High moderate & average certainty (greater than 50%)	High moderate & average certainty (greater than 50%)	[illegible]
**Availability of treatment**	Treatments that cure conditions or manage their symptoms	Treatments that cure conditions or manage their symptoms	[illegible]
Additional number of newborns diagnosed compared to standard [illegible] ed)	50 in 1000	20 in 1000	[illegible]
[illegible]	100% (none of the newborns screened will receive a wrong initial diagnosis)	95% (5 out of 100 newborns screened will receive a wrong initial diagnosis)	
[illegible] ning to	$2,500	$1000	$0

○ Genomic Newborn Screening 1 ○ Genomic Newborn Screening 2 ○ Standard Newborn Screening only

Both include conditions with **high, moderate & average** certainty, which means that conditions **with as low as 50% certainty** that they develop are included in the screening program.

Both include conditions where treatments that **cure conditions or manage their symptoms** are included.
This means that for every child diagnosed through the screening there is a treatment available that can improve their life or even cure their health condition.

Example 1:

	Genomic Newborn Screening 1	Genomic Newborn Screening 2	Standard Newborn Screening only
**Severity of the condition**	Profound moderate & mild	Profound & moderate	
**Certainty that the condition develops**	High moderate & average certainty (greater than 50%)	High moderate & average certainty (greater than 50%)	
**Availability of treatment**	Treatments that cure conditions or manage their symptoms	Treatments that cure conditions or manage their symptoms	
**Additional number of newborns diagnosed compared to standard newborn screening (in every 1000 newborns screened)**	50 in 1000	20 in 1000	
**Accuracy of scr[illegible]ning results**	100% (none of the newborns screened will receive a wrong initial diagnosis)	95% (5 out of 100 newborns screened will receive a wrong initial diagnosis)	
[illegible]	$2,500	$1000	$0
	○ Genomic Newborn Screening 1	○ Genomic Newborn Screening 2	○ Standard Newborn Screening only

Genomic Newborn Screening 1 diagnoses **50** additional newborn relative to the Standard Newborn Screening. These are diagnoses that would have been missed otherwise.

Genomic Newborn Screening 2 can find **20** additional diagnoses compared to Standard Newborn Screening.

Example 1:

	Genomic Newborn Screening 1	Genomic Newborn Screening 2	Standard Newborn Screening only
Severity of the condition	Profound moderate & mild	Profound & moderate	
Certainty that the condition develops	High moderate & average certainty (greater than 50%)	High moderate & average certainty (greater than 50%)	
Availability of treatment	Treatments that cure conditions or manage their symptoms	Treatments that cure conditions or manage their symptoms	
**Additional number of newborns diagnosed compared to standard newborn screening (in every 1000 newborns screened)**	50 in 1000	20 in 1000	
Accuracy of screening results	100% (none of the newborns screened will receive a wrong initial diagnosis)	95% (5 out of 100 newborns screened will receive a wrong initial diagnosis)	
	$2,500	$1000	$0

Genomic Newborn Screening 1 can diagnose **30 more** newborns compared to Genomic Newborn Screening 2, because it also screens for **mild conditions**.

This means it can make more diagnoses because it looks for more conditions than Genomic Newborn Screening 2.

○ Genomic Newborn Screening 1

○ Genomic Newborn Screening 2

○ Standard Newborn Screening only

Example 1:

For Genomic Newborn Screening 1 **100% of screening results are correct**, which means all newborns labelled as 'high chance' will indeed have the condition.

For Genomic Newborn Screening 2 **95% accurate** means that out of every 100 newborns labelled as 'high chance', 95 of them will indeed have the condition while **5 will not.**

	Genomic Newborn Screening 1	Genomic Newborn Screening 2	Standard Newborn Screening only
[illegible]	Profound moderate & mild	Profound & moderate	
[illegible]elops	High moderate & average certainty (greater than 50%)	High moderate & average certainty (greater than 50%)	
[illegible]	Treatments that cure conditions or manage their symptoms	Treatments that cure conditions or manage their symptoms	
[illegible]s diagnosed compared to standard newborn screening (in every 1000 newborns screened)	50 in 1000	20 in 1000	
**Accuracy of screening results**	100%<br>(none of the newborns screened will receive a wrong initial diagnosis)	95%<br>(5 out of 100 newborns screened will receive a wrong initial diagnosis)	
Cost of genomic newborn screening to you	$2,500	$1000	$0

I would prefer:

○ Genomic Newborn Screening 1

○ Genomic Newborn Screening 2

○ Standard Newborn Screening only

Example 1:

	Genomic Newborn Screening 1	Genomic Newborn Screening 2	Standard Newborn Screening only
Severity of the condition	Profound moderate & mild	Profound & moderate	
[illegible]	High moderate & average certainty (greater than 50%)	High moderate & average certainty (greater than 50%)	
[illegible]	Treatments that cure conditions or manage their symptoms	Treatments that cure conditions or manage their symptoms	
[illegible]	50 in 1000	20 in 1000	
[illegible]	100% (none of the newborns screened will receive a wrong initial diagnosis)	95% (5 out of 100 newborns screened will receive a wrong initial diagnosis)	
**Cost of genomic newborn screening to you**	$2,500	$1000	$0
**I would prefer:**	◯ Genomic Newborn Screening 1	◯ Genomic Newborn Screening 2	◯ Standard Newborn Screening only

For Genomic Newborn Screening 1 you would have to pay **$2,500**.
For Genomic Newborn Screening 2 you would have to pay **$1,000**.

This means you would have to pay more for Genomic Newborn Screening 1.

You would not have to pay anything for Standard Newborn Screening.

Example 1:
Now compare Genomic Newborn Screening 1 and Genomic Newborn Screening 2.
Your task:

- Imagine you have a newborn and are offered the opportunity to take part in a genomic newborn screening program.
- Choose the newborn screening scenario that you would like the most for your child.
- If you would not choose either of the genomic newborn screening programs, select the option 'no genomic screening'. Your child would still have the standard 'heel prick test' that checks for 25 rare but serious childhood conditions. Since this is a public health initiative, you would not need to pay anything for standard newborn screening.

Which of the options would you choose? Click on your choice below.

	Genomic Newborn Screening 1	Genomic Newborn Screening 2	Standard Newborn Screening only
**Severity of the condition**	Profound moderate & mild	Profound & moderate	
**Certainty that the condition develops**	High moderate & average certainty (greater than 50%)	High moderate & average certainty (greater than 50%)	
**Availability of treatment**	Treatments that cure conditions or manage their symptoms	Treatments that cure conditions or manage their symptoms	
**Additional number of newborns diagnosed compared to standard newborn screening (in every 1000 newborns screened)**	50 in 1000	20 in 1000	
**Accuracy of screening results**	100%<br>(none of the newborns screened will receive a wrong initial diagnosis)	95%<br>(5 out of 100 newborns screened will receive a wrong initial diagnosis)	
**Cost of genomic newborn screening to you**	$2,500	$1000	$0

I would prefer:

○ Genomic Newborn Screening 1

○ Genomic Newborn Screening 2

○ Standard Newborn Screening only

We will ask you to make 9 choices like this.

For this task, please imagine you have a newborn and are offered the opportunity to take part in a genomic newborn screening program.

- Please choose the newborn screening program that you would like the most for your child.
- If you would not choose either of the genomic newborn screening programs, please select the option 'no genomic screening'. Your child would still have the standard 'heel prick test' that checks for 25 rare but serious childhood conditions. Since this is a public health initiative, you would not need to pay anything for standard newborn screening.
- Some of these characteristics will be greyed out in each situation, this means they are the same across both options. Before choosing, please ensure that you have considered all characteristics. It is important that you consider not only the characteristics that are different but also the ones that are common between the two situations.
- There are no right or wrong answers.
- In surveys like this, it has been found that some people tend to overestimate or underestimate how much they would really be willing to pay. So, they may choose situations they would not prefer in real life. It is important that your choices here are realistic.
- If you would like to remind yourself during the survey what each of these characteristics means, point/click on the characteristic of interest, and a pop-up box with the description will appear.

Feedback

How did you find the questions in this survey?

☐ Easy ☐ Moderate ☐ Difficult

Have you got any comments about this questionnaire that you would like to share with us?

____

____

____

Acknowledgement

Thank you for taking the time to complete our survey. We really appreciate your time and input!

THE UNIVERSITY OF MELBOURNE

Your say on the process of Genomic Newborn Screening

Can you take part in our survey?

We want to hear your thoughts about genomic newborn screening. Newborn screening programs in Australia do not use genomic testing at present. But there is potential for it to be used in the future. We want you to have a say on what this may look like. We have developed a survey that takes about 20 minutes to complete. You'll start by watching a quick video on genomic newborn screening. Afterward, we'll present you with a series of questions where you can choose between different genomic newborn screening programs, so that we can learn about what aspects are important to you. We will also ask for some information about you, like your age and gender.

What is newborn screening?

Every new baby has a test to screen for genetic conditions at birth. This is performed on a few drops of blood collected soon after birth by pricking the baby's heel, called the 'heel prick test'. Newborn screening looks for around 25 rare but serious health conditions in babies. These conditions can be life-threatening or affect development.

What is genomic newborn screening?

New technologies can be used in newborn screening to look for many more health conditions. One of these is genomic testing. This is a powerful tool we use to look for DNA changes that can impact health. To see these changes, we map out a person's whole genetic code. The DNA changes we find can reveal useful information about the chance of developing one or many different health conditions. It is possible to screen for many treatable childhood-onset conditions at once using genomic testing. This is called genomic newborn screening

Taking part in this survey is voluntary. Your responses will be confidential, not identifiable and not shared with anyone outside the research team. This research has ethical approval from The Royal Children's Hospital Melbourne. You can withdraw from this research at any time and without any consequences.

Please click here to review the **Participant Information Statement**, where we describe the project and what taking part involves.

If you have any questions, please contact Dr Riccarda Peters or A/Professor Ilias Goranitis.

Thank you for your time and help! We value your input. Your thoughts will be used to inform future genomic screening programmes.

The consent process

By clicking the arrow to proceed, I am indicating my consent to participate in this study as outlined in the Participant Information Statement.

Screening questions:

Before you proceed to the survey, we need to confirm you meet all the criteria below to take part. Please click the appropriate button below:

1. I am aged 18 or older

☐ Yes

☐ No

2. I am an Australian citizen or resident

☐ Yes

☐ No

3. I speak English

☐ Yes

☐ No

(*No to any 1-3 do not qualify*)

Demographics

About you

We will ask a few questions about you so we can better understand your answers.

1. How old are you? __________________ years

2. What is your gender:

☐ Female
☐ Male
☐ Prefer to self-describe __________________

3. What state or territory do you live in? Please check only one.

☐ New South Wales
☐ Victoria
☐ Queensland
☐ South Australia
☐ Western Australia
☐ Tasmania
☐ Northern Territory
☐ Australian Capital Territory

4. What is your highest level of education?

☐ Year 11 or below
☐ Year 12 or equivalent
☐ Certificate
☐ Diploma/advanced diploma
☐ Bachelor's degree
☐ Graduate diploma/certificate
☐ Post-graduate degree
☐ Other (please specify): __________________

5. What is your household's gross annual income – that is, your earnings before tax?

☐ Lower than $40,000 per year
☐ $40,000 - $60,000 per year
☐ $60,000 - $80,000 per year
☐ $80,000 - $100,000 per year
☐ $100,000 - $120,000 per year
☐ $120,000 - $140,000 per year
☐ $140,000 - $160,000 per year
☐ Over $160,000 per year

6. Do you have private health insurance?

☐ Yes
☐ No

7. What is your current marital status?

☐ Never married
☐ De facto living with a partner
☐ Married
☐ Widowed
☐ Divorced / separated
☐ Other (please specify): __________________

8. Do you have children?

☐ Yes (if yes, go to 9)
☐ No (if no, go to 11)

9. How many children do you have? _______
10. How old is your youngest child? Age in years _______

11. Are you or your partner currently pregnant?

☐ Yes
☐ No
☐ Prefer not to say
☐ Not applicable

12. Do you plan to have children in the near future? Please check only one.

☐ Yes
☐ No
☐ Not sure
☐ Prefer not to say

Experience with genetic conditions and newborn screening

13. A genetic condition is a health issue caused by changes in our DNA that can affect how our bodies work. Do you or any of your close family members or friends have a genetic condition?

☐ Yes
☐ No
☐ Do not know

14. A genetic or genomic test is a test that looks at our DNA to find out information about our genes and how they might affect our health. Have you or any of your close family members or friends ever had a genetic or genomic test?

☐ Yes
☐ No
☐ Do not know

15. Have you read or heard about genomic testing before receiving this survey?

☐ Yes
☐ No

16. How familiar do you feel with how genetic conditions affect people's lives and the lives of those around them?

Give a number between 0- 10. Here, 0 means 'I am not familiar at all'. And 10 means 'I know exactly what it means to have a genetic condition or to live with someone who has a genetic condition':

____

17. How much did you know about newborn screening before receiving the invitation to participate in this survey? Please check only one.

☐ I had never heard of newborn screening
☐ I had heard of newborn screening, but I did not know anything about it
☐ I knew a little about newborn screening
☐ I knew a lot about newborn screening

Attitudes towards risk

18. In general, people often face risks when making health, financial, career and other life decisions. Overall, how comfortable do you feel you are in taking risks regarding your health?

Give a number between 0- 10. Here, 0 means 'I am not comfortable at all'. And 10 means 'I am extremely comfortable':

____

Knowledge about health

19. How often do you need to have someone help you when you read instructions, pamphlets, or other written material from your doctor or pharmacy?
(5-point Likert-type scale, 1 = never to 5 = always)

Background

Please watch the short video below to learn more about newborn screening and genomic newborn screening.

https://player.vimeo.com/video/834581569

Genomic testing is not used in newborn screening in Australia at the moment. But it may be used in the future. What exactly this will look like is uncertain, this is why we need your help.
The data we get from genomic newborn screening is not the same as what we get from standard newborn screening. Because of this, there are extra things to think about when using genomics in a newborn screening program. This survey is about what you think is important to consider for genomic newborn screening.

Concerns regarding the use of genomics in newborn screening programs

Using genomics in newborn screening poses issues that we need to consider.

- Genomic data are challenging to understand and interpreting them requires special knowledge.
- Analysing genomic data is complex, and misinterpretation could lead to the wrong diagnoses or treatments.
- Genomic data is sensitive; some may worry it could affect things like insurance and jobs when the child grows up. In Australia, health insurers are generally prohibited from asking for or using genetic test results for most types of insurance. However, in certain circumstances life insurers in Australia can request and use genomic test results. Insurance regulations can also change over time, and there may be variations in state or territory laws.
- Some people may not have as much access to genomic screening, which could make health inequalities between different groups more significant.

Genomic newborn screening characteristics

In this section, we will go over eight things that are important when thinking about genomic newborn screening programs. This information is not typically included in the regular tests done during pregnancy. Make sure to read this carefully because in the next part, we will ask you to make choices based on how much these characteristics matter to you.

Characteristic descriptions:

1. When will genomic newborn screening first be discussed?

This characteristic tells you when future parents will hear about genomic newborn screening for the first time. There are many important tests being done during pregnancy (as you can see in the timeline below) and information about genomic newborn screening is separate to all of these.
If you get information about genomic newborn screening early in pregnancy, it gives you time to think and decide what's best for you. But it might feel like a lot to deal with when there are so many other things to think about during pregnancy. Some parents might prefer to get this information closer to when the baby will be tested once they are born, so it's fresh in their minds and doesn't get mixed up with other things.

The options are:

- **<u>Early during pregnancy at your first doctor appointment</u>**
- **<u>Second trimester</u>**
- **<u>Third trimester</u>**
- **<u>Shortly after birth</u>**

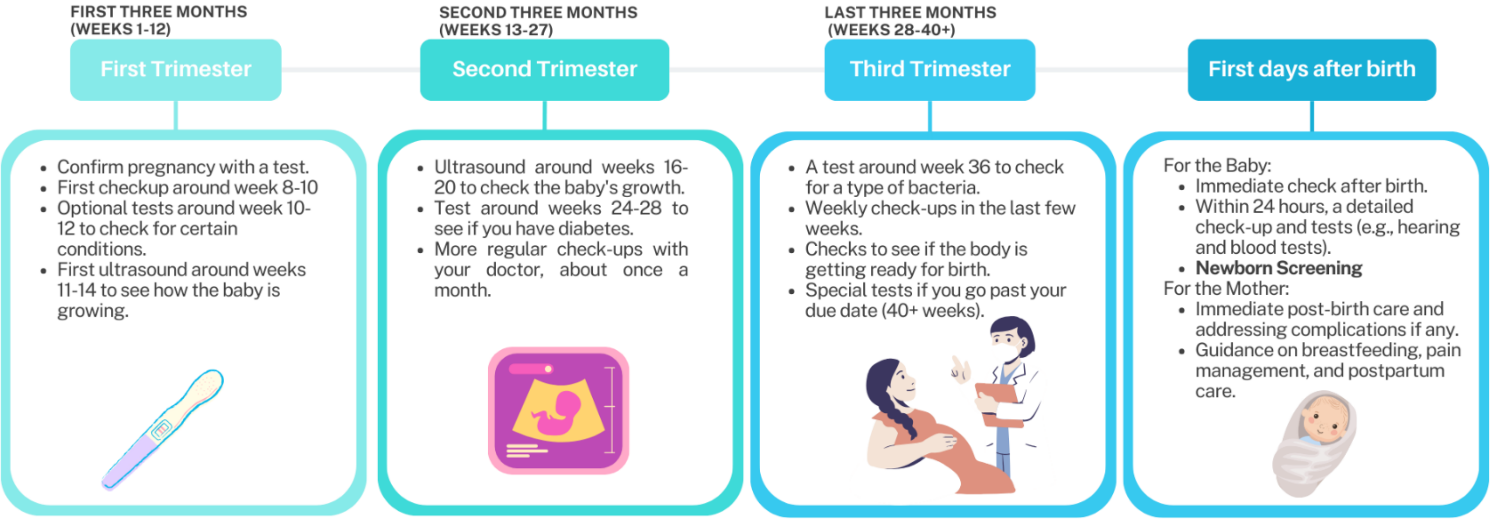

THE UNIVERSITY OF MELBOURNE

2. Who provides initial information about genomic newborn screening?

This characteristic tells you who will give parents the first details about genomic newborn screening.

The options are:

- **A midwife or nurse:** Some people may prefer receiving information about the screening program from a midwife or nurse because they tend to be seen as approachable, empathetic, and have experience explaining complex medical issues in simple terms.
- **Your GP (General Practitioner):** GPs are often a family's primary point of contact for healthcare. Families trust their GP's expertise and rely on them for general health guidance. They may feel more comfortable discussing sensitive topics like genomic newborn screening with a healthcare provider they know well. However, GPs don't have specialist expertise in genomic newborn screening.
- **An obstetrician:** Some people like to get information about newborn screening from obstetricians. Obstetricians are experts in prenatal care, they will build a relationship with the parents during pregnancy, and they can provide personalized care for the family. They make sure discussions about screening are part of overall pregnancy care.
- **A genetic health professional:** Other people may prefer to discuss genomic newborn screening with a genetic health professional because they are specially trained in human genetics, counselling, and making sure their patients understand everything clearly.

3. What support material is available about genomic newborn screening?

When parents learn about genomic newborn screening, they might receive extra materials in addition to what is discussed in their first information session to help them understand what it is and how it works. They can get this extra information in different ways.

The options are:

- **No support material:** Parents will not receive any additional support material aside from what is discussed in the first information session with the midwife/nurse/GP/genetic health professional.
- **Leaflet:** Parents will receive a concise leaflet that provides an overview and essential details about genomic newborn screening. Think of it as a brief introductory guide.
- **Interactive online portal:** Parents will be given a link to a dedicated website about the newborn screening program. There, they will find comprehensive resources, including decision aids and informative videos.
- **Appointment:** Parents will have the opportunity to set up a dedicated appointment with a health professional to discuss genomic newborn screening with them and answer any further questions they have.

4. What conditions are included in genomic newborn screening?

Genomic newborn screening offers the potential to investigate hundreds of conditions at once, and parents may or may not be able to choose which conditions they would like their newborn to be screened for.

The options are:

- **Conditions selected by parents:** The parents will receive information on conditions grouped according to categories. The categories could be based on things like how serious the condition is, when it might show up, or whether there are treatments available. The parents get to decide which groups of conditions they want their baby to be tested for.
- **Conditions selected by health professionals:** Parents will not have a choice about which conditions their baby will be screened for. Doctors and scientists have prepared a list of conditions based on the best clinical evidence, and all babies will be screened for the same conditions.

5. Who returns "high chance" results from genomic newborn screening?

The baby's test results may show they have a high chance of having a genetic condition. Genetic conditions are very rare and results can be complex. This characteristic tells you who returns the test results to the parents if their baby is identified to have a high chance for a genetic condition through genomic newborn screening.

The options are:

- **Your GP (General Practitioner):** Some people prefer to receive the results from their general practitioner because they feel more comfortable discussing personal information with them. However, GPs don't have specialist expertise in rare genetic conditions.
- **A relevant medical specialist:** Other people may prefer to receive the results from a relevant medical specialist who works in the area related to the condition that has been identified, as they are more interested in the medical implications of their results.
- **A genetic health professional:** Some people prefer to receive genomic results from a genetic health professional because of their expertise in genomic sequencing, their ability to interpret the results in a clear way, and experience in supporting people through the process of receiving genomic results.

6. How are "high chance" results from genomic newborn screening returned?

In cases where the results of the genomic newborn screening are "high chance", this characteristic tells you how parents will receive these results. This can be directly from a health professional or electronically through a secure online portal.

The options are:

- **Electronically through a secure online portal:** This would allow parents to see the results as soon as they are available and gives them control over their own results, including who to discuss them with and when. However, the results may be distressing, and parents may feel like they need more support.
- **Directly, telehealth or phone:** Parents receive results directly from a health professional via telehealth or phone. This allows parents to ask questions and get a good understanding of genomic newborn screening results. However, they do not have instant access to the results but have to wait until their appointment with the health professional which will be via telehealth or phone and not in person. Some people may prefer this as they will not have to travel to an appointment.
- **Directly, in person:** Parents receive results directly from a health professional in person. This allows parents to ask questions and get a good understanding of genomic newborn screening results. They would not have instant access to the results but have to wait until their appointment with the health professional. This means they will have to travel to an appointment.

7. How are "low chance" results from genomic newborn screening returned?

When newborn screening doesn't detect any genetic changes to indicate the medical problems screened for, this is called a "low-chance result". In the standard newborn screening program, parents do not receive 'low chance' results.

If the genomic newborn screening returns a low chance result, parents may receive this directly from a health professional or through a secure online portal.

The options are:

- **No return of "low chance" results:** Like in the standard newborn screening program, parents will not be contacted if the screening does not detect any genetic changes to indicate the medical problems screened for.
- **Electronically through a secure online portal:** "Low chance" results will be made available in a secure online portal. This would allow parents to see the results as soon as they are available and gives them control over their own results, including who you discuss them with and when. Parents may not be sure how to interpret "low chance" results and may feel like they need more support.

- **Directly, telehealth or phone:** Parents receive results directly from a health professional via telehealth or phone. This allows parents to ask questions and get a good understanding of what it means to have a "low chance" result. However, they do not have instant access to the results but have to wait until their appointment with the health professional which will be via telehealth or phone and not in person. Some people may prefer this as they will not have to travel to an appointment.
- **Directly, in person**: Parents receive results directly from a health professional in person. This allows parents to ask questions and get a good understanding of what it means to have a "low chance result". They would not have instant access to the results but have to wait until their appointment with the health professional. This means they will have to travel to an appointment.

8. What happens if new relevant information about genomic newborn screening becomes available?

Over time, new information may become available related to the baby's results from genomic newborn screening. For example, more information relevant to the baby or their family's health may become available because of improvements in knowledge or changes in clinical practice. This characteristic tells you what happens if new relevant information about genomic newborn screening becomes available. Initially when genomic newborn screening will be introduced, no updates will be provided automatically. However, there is an opportunity to provide an update upon individual request later or to provide ongoing updates through a secure online portal.

The options are:

- **No updates will be provided**
- **Updates will be provided upon request:** Updates will not be sent automatically, if parents are interested in receiving updates, they would have to request them.
- **Updates will be provided automatically in a secure online portal:** Parents will have access to a secure online portal where updates will be automatically provided when they become available.

Consent

Consent for genomic newborn screening means that the newborn's parent(s) agree to the screening on behalf of the baby. This means they understand what the test does, why it is needed, and what might be good or not so good about it. There are a few ways to give permission:

- Opt-in: This is when parents have to actively say "yes" by signing a form or checking a box to agree to the test.
- Opt-out: Here, parents are considered to agree unless they say "no." It means you are automatically included unless you say you don't want it.
- Implied consent: This is when people understand that the test is just a routine part of healthcare, so they don't need to say "yes" explicitly. By bringing their baby, they are agreeing to it, and all babies get tested.

Which method for giving consent would you prefer for genomic newborn screening?

a) Opt-in
b) Opt-out
c) Implied consent

DCE explanation and example

In this section, we'll present two genomic screening programs side by side. You can see an example below. These programs consist of the eight features we discussed earlier, and you can compare them.

Your task:

- Please imagine you have a newborn and are offered the opportunity to take part in a genomic newborn screening program.
- We ask you to compare these genomic newborn screening programs based on four out of the eight characteristics. The specific four characteristics may vary. For the remaining characteristics, assume they are equal between the two programs.
- Please choose the newborn screening program that you would like the most for your child.
- You can choose "Neither" if you prefer neither option.

Example:

Which screening program do you prefer?

	**Genomic Newborn Screening 1**	**Genomic Newborn Screening 2**
**When** is genomic newborn screening first discussed?	Early during pregnancy at first doctor appointment	Shortly after birth
**Who** returns "high chance" results?	Your GP	A genetic health professional
**How** are "high chance" results returned?	Electronically through a secure online portal	Directly, in person
What happens if **new relevant information** becomes available?	Updates will be provided upon request	No updates will be provided

I would choose:

☐ **Genomic Newborn Screening 1**

☐ **Genomic Newborn Screening 2**

☐ **Neither**

DCE explanation and example

Now, let's go over an example together before we start the task.

The situation below has two genomic newborn screening programs ('Genomic newborn screening 1' and 'Genomic newborn screening 2') that are a bit different in four out of the eight characteristics we talked about before.

Which newborn screening program would you choose?

You have three options:

"**Genomic newborn screening 1**," "**Genomic newborn screening 2**," or "**Neither**".

	Genomic Newborn Screening 1	Genomic Newborn Screening 2
**When** is genomic newborn screening first discussed?	Early during pregnancy at first doctor appointment	Shortly after birth
**Who** returns "high chance" results?	Your GP	A genetic health professional
**How** are "high chance" results returned?	Electronically through a secure online portal	Directly, in person
What happens if **new relevant information** becomes available?	Updates will be provided upon request	No updates will be provided

I would choose:

✖	☐	☐
**Genomic Newborn Screening 1**	**Genomic Newborn Screening 2**	**Neither**

The person who answered this question picked "Genomic Newborn Screening 1." They examined the differences between "Genomic Newborn Screening 1" and "Genomic Newborn Screening 2". The newborn screening processes differed in when genomic newborn screening should be discussed, who would return "high chance" results and how these would be returned, and what happens when new relevant information about genomic newborn screening becomes available. This person liked the profile of "Genomic Newborn Screening 1" better.

We will ask you to make 8 choices like this.

For this task, please imagine you have a newborn and are offered the opportunity to take part in a genomic newborn screening program.

- Please choose the newborn screening program that you would like the most for your child.
- Before choosing, please ensure that you have considered all characteristics.
- There are no right or wrong answers.

Feedback

How did you find the questions in this survey?

☐ Easy ☐ Moderate ☐ Difficult

Have you got any comments about this questionnaire that you would like to share with us?

____

____

____

Acknowledgement

Thank you for taking the time to complete our survey. We really appreciate your time and input!
